# Supplementary material for: Bayesian spatio-temporal modeling of malaria risk in Rwanda
Source: PLoS One. 2020 Sep 10;15(9):e0238504. doi: 10.1371/journal.pone.0238504 (PMC7482939; doi:10.1371/journal.pone.0238504)
Supplement: Attachment — Submitted filename: PONE-D-19-30728.pdf [file pone.0238504.s004.pdf]

Review comments

The paper addresses an important issue on using population data and clinical routine malaria data for decision making in control/elimination of malaria. The paper uses a model based approach in the analysis and mapping of malaria incidence in Rwanda, for the period from 2012 to 2018.

A) Minor comments

a. Abstract: specify the SDG number that is being referred to there.
b. Abstract: "The results showed an increase of risk of malaria and 47.36% of sectors in Rwanda" is not very clear. Is this increase national or in specific sectors only?
c. Line 9:10, do the authors mean "categorised" when they mention "situated"?
d. Line 14: Most countries "placed"….
e. Line 36 – 37: …finally increasing %... use "percentage" instead of the symbol.
f. Line 45: For the "epidemiological"….
g. Line 46 – 47: Re-write to make it clear.
h. Line 54 – 55: …. Patterns in low areas transmission should change to …. "patterns in low transmission areas".
i. Line 91 – 92: Can authors specify what percentage of the total was not included in the final analysis?
j. Line 97 – 102: Authors need to indicate by properly subscripting the count for time as rightly done for geographic areas in the same paragraph.
k. In Table 1, can the authors add footnotes (or in the caption) to explain what SD, LL and UL are?
l. Line 228: Change "sound statistics" to "accurate statistics".
m. Line 234 – 236 is not clear in the current form.
n. Line 237: This in the current form is misleading. Should it be reading: .... almost half (47.36%) of all sectors....
o. On lines 246 – 249, what threshold is being looked at here? It's not coming out very clearly.
p. Figures are not (properly) captioned, making it difficult to follow or align the text to the Figures.
    i. The Figure (Fig1_Desc2), titled "The under-five Malaria 2012 0 2018" with no sex specified seems redundant as it is adding very little information. The information presented in the graph can be explained in the text.
    ii. The Y-axis on Fig1_Desc5 "Overall malaria per year" is misleading. The Figure needs to be re-done to properly convey the correct information.
    iii. The legends in Figures 2 – 4 should be properly positioned, interfering with maps currently.
q. Go through the manuscript to correct typos and grammatical errors.

B) Major comments

a. The language in the current form of the paper still needs extensive editing to make things clear.

   i. Abbreviations are not properly defined throughout the document i.e. figure 3 instead of Figure 3 etc.
   ii. Capitalisation is not properly used throughout the document.
   iii. Several sentences are not very clear as indicated in the minor comments above.
   iv. Several places missing commas and full stops – distorting the message
   v. Inappropriate tenses used.
   vi. Inappropriate use of directions i.e. "east north" as indicated on lines 237 - 250 instead of "North east"

b. Lines 119 – 122: Authors indicate that they use Bayesian methods for the analysis, and. Have taken the time to explain both the data and process models. However, conspicuously missing are details on the priors used in model fitting.

c. Line 132 – 136: Authors introduce the concept of calculating policy relevant threshold. Three issues arise here.

   i. How is the threshold "*c*" determined or reached at? This is not clearly explained in the document. For the reader to understand the policy relevant goals, determination of these thresholds needs to be clearly explained.
   ii. In line 135, authors claim that: "If |P| is large, the set goal is likely reached in that area." What is |P| being compared to?
   iii. In P(Theta_$_{it}$ < (100 - c)%), is the relative risk based on the observed data? If so, this has to be explicitly stated.

d. The authors have 7 years worthy of data. Enough data to enable a proper spatio-temporal model. However, in lines 157 – 159, they indicate that they have fitted separate models for each year. I find this problematic in the sense that they are underutilising the data and not fully leveraging the information therein. A spatio-temporal model will enable borrowing strengths in the data across both space and time, therefore giving a complete picture of how malaria incidence has changed since the base year of 2012. The model fitted currently has huge implications on the conclusions authors draw in the paper. I comment on this in later sections below.

e. In Tables 2 and 4 and lines 174 – 192, authors present malaria relative risk per year as compared to base year of 2012. They group RR into 5 groups: 0-1, 1 – 4, 4 – 10, 10 – 15, and 15 – 24. They use square braces. This presents several challenges in the sense that:

i. The mathematical/statistical meaning of these braces means that these groups are not distinct, meanwhile in text, these groups of RR are presented as distinct. Authors should not [0, 1] and [0, 1) will mean two different things.
ii. Again, for example, column one has [0, 1] and column two has [1, 4]. Does this mean that these two RR groups contain both the 1? This is same for all the groups and it is misleading.
iii. More confusing is the fact that in the text, they resort to using round braces. For reasons in point 1 above, this becomes more confusing.
iv. Therefore lines 184 – 192 need to be re-written with correct presentation of Table 2.

f. In section 3.3, lines 193 - 215 authors present an "assessment of Malaria policy to reduce incidence in Rwanda." With this goal of analysis in mind, it makes more sense to use spatio-temporal model, so that the **available data take into account** the trends leading up to the target year (2015 and 2018) for the target non-exceedance thresholds of 20% and 42% respectively. See comment in point (d) above.

g. Lines 203 – 205: The authors should endeavor to quantify this increase, for it to be helpful and relevant to policy makers.

h. Lines 206 – 209 should be re-written to properly convey the message contained in there. More importantly, the claims raised in these lines can be affirmed by using a proper spatio-temporal analysis in relation to the concerns raised in points (d) and (f) above.

i. Line 237 – 239. Authors claim that almost half (47.36%) of the sectors did not meet the targets with 80 or 90% certainty. What would be helpful is for the authors to show clearly each of these certainties on map. See, for example:
i. Giorgi et. al. (2018), Using non-exceedance probabilities of—relevant malaria prevalence thresholds to identify areas of low transmission in Somalia. Malar J. 2018;17:88.

ii. Macharia et. al. (2019), Spatio-temporal analysis of *Plasmodium falciparum* prevalence to understand the past and chart the future of malaria control in Kenya

iii. Yankson et. al. (2019), Geostatistical analysis and mapping of malaria risk in children under 5 using point-referenced prevalence data in Ghana

j. In their discussion, on lines 250 – 251, authors mention that “Implementing pre-elimination strategies in those sectors should be considered consciously.” With the the incidence presented here, it’s not proper for the authors to start talking about pre-elimination. The message to policy implementers should rather be to focus on control strategies at this point.

k. On lines 258 – 259, authors mention that “It can contribute to improve Malaria surveillance to ensure appropriate intervention in the right place and most needed time.” This is very correct, but based on the statistical analysis presented here, authors should be cautious in their statements on conclusions made. A proper spatio-temporal analysis would be required to make this conclusion on time.
