# Supplementary material for: Bayesian spatio-temporal modeling of malaria risk in Rwanda
Source: PLoS One. 2020 Sep 10;15(9):e0238504. doi: 10.1371/journal.pone.0238504 (PMC7482939; doi:10.1371/journal.pone.0238504)
Supplement: Attachment — Submitted filename: PONE-D-19-30728_R1.pdf [file pone.0238504.s006.pdf]

I appreciate the opportunity to review this revised paper. The authors have resolved some of the initial problems in the analysis, particularly the issue of using an appropriate modelling approach for the kind of data and objectives of analysis at hand. There are however still a few issues remaining.

a. Language in the revised form of the paper still falls shy of the required standard. As advised previously, several things are still glaringly evident such as, among others. I strongly advise the authors to seek services of a professional editing service.
    i. Several places missing commas and full stops – distorting the message
    ii. Inappropriate tenses used.
    iii. Inappropriate use of directions i.e. “east north” as indicated on instead of “North east”.
b. Lines 70 – 71 are confusing, the authors indicate that they use spatial disease mapping techniques, whereas in in methods, they indicate that they are using spatio-temporal methods.
c. Lines 145 – 147 What is the justification for using the gamma prior Ga(0.5, 0.00149) apart from just copying what Fong et. al. did?
d. Table 2, the authors present a number of things including DICc and WAIC, which they don’t discuss anywhere in the text. Explain what these are.
e. Tables 4 and 5, the headers for the Table are wrongly placed, the braces are wrong as well, edit to correct.
f. There is a disconnect between the paper’s discussion and methods used, hence results found – methods aren’t discussed to highlight how the results and conclusions made have been reached at.
g. I attach the PDF file on which I made a number of comments that will be helpful to the authors to sort out the highlighted issues.
