# Supplementary material for: Bayesian spatio-temporal modeling of malaria risk in Rwanda
Source: PLoS One. 2020 Sep 10;15(9):e0238504. doi: 10.1371/journal.pone.0238504 (PMC7482939; doi:10.1371/journal.pone.0238504)
Supplement: Attachment — Submitted filename: PONE-D-19-30728_R1_reviewer_comments.pdf [file pone.0238504.s007.pdf]

PLOS ONE

Bayesian spatio-temporal modeling of malaria risk in Rwanda

--Manuscript Draft--

**Manuscript Number:**	PONE-D-19-30728R1
**Article Type:**	Research Article
**Full Title:**	Bayesian spatio-temporal modeling of malaria risk in Rwanda
**Short Title:**	Bayesian spatio-temporal modeling of malaria risk in Rwanda
**Corresponding Author:**	Muhammed Semakula<br>University of Rwanda College of Business and Economics<br>Kigali, City of Kigali RWANDA
**Keywords:**	Spatio-temporal models, Malaria risk
**Abstract:**	Every year, 435,000 people worldwide die from Malaria, mainly in Africa and Asia. However, malaria is a curable and preventable disease. Most countries are planning malaria elimination to meet sustainable development goal three, target 3.3, of ending malaria by 2030. Rwanda, through the malaria strategic plan 2012-2018 set a target to reduce malaria incidence by 42% from 2012-2018. Assessing the health policy and taking a decision using the incidence rate approach is becoming more challenging. We are proposing suitable statistical methods that handle spatial structure and uncertainty on the relative risk that is relevant to National Malaria Control Program.<br>We used spatio-temporal model to estimate the excess probability for decision making at a small area on evaluating reduction of incidence. SIR and BYM models were developed using Health facilities routine data from 2012-2018 in Rwanda. The fitted model was used to generate relative risk (RR) estimates comparing the risk with malaria risk in 2012, and to assess the probability of attaining the set target goal per area.<br>The results showed an overall increase in malaria in 2016 particularly. The 47.36% of all sectors in Rwanda failed to meet the target reduction from 2012 to 2018. Our approach of using excess probability method to evaluate attainment of target or identifying threshold is a relevant statistical method, which will enable the Rwandan Government to sustaining malaria control and monitoring targeted interventions.
**Order of Authors:**	Muhammed Semakula
	FranÇois Niragire
	Christel Faes
**Response to Reviewers:**	Response to the reviewers are attached in separate letter
**Additional Information:**	
**Question**	**Response**
**Financial Disclosure**<br><br>Enter a financial disclosure statement that describes the sources of funding for the work included in this submission. Review the submission guidelines for detailed requirements. View published research articles from *PLOS ONE* for specific examples.<br><br>This statement is required for submission and **will appear in the published article** if the submission is accepted. Please make sure it is accurate.	The authors received no specif funding for this work.

Powered by Editorial Manager® and ProduXion Manager® from Aries Systems Corporation

**Unfunded studies**<br>Enter: *The author(s) received no specific funding for this work.*<br><br>**Funded studies**<br>Enter a statement with the following details:<br>• Initials of the authors who received each award<br>• Grant numbers awarded to each author<br>• The full name of each funder<br>• URL of each funder website<br>• Did the sponsors or funders play any role in the study design, data collection and analysis, decision to publish, or preparation of the manuscript?<br>• **NO** - Include this sentence at the end of your statement: *The funders had no role in study design, data collection and analysis, decision to publish, or preparation of the manuscript.*<br>• **YES** - Specify the role(s) played.<br><br>* typeset	
**Competing Interests**<br><br>Use the instructions below to enter a competing interest statement for this submission. On behalf of all authors, disclose any competing interests that could be perceived to bias this work—acknowledging all financial support and any other relevant financial or non-financial competing interests.<br><br>This statement **will appear in the published article** if the submission is accepted. Please make sure it is accurate. View published research articles from *PLOS ONE* for specific examples.	The authors have declared that no competing interests exist

**NO authors have competing interests**<br><br>Enter: *The authors have declared that no competing interests exist.*<br><br>**Authors with competing interests**<br><br>Enter competing interest details beginning with this statement:<br><br>*I have read the journal's policy and the authors of this manuscript have the following competing interests: [insert competing interests here]*<br><br>* typeset	
**Ethics Statement**<br><br>Enter an ethics statement for this submission. This statement is required if the study involved:<br><br>• Human participants<br>• Human specimens or tissue<br>• Vertebrate animals or cephalopods<br>• Vertebrate embryos or tissues<br>• Field research<br><br>Write "N/A" if the submission does not require an ethics statement.<br><br>General guidance is provided below. Consult the submission guidelines for detailed instructions. **Make sure that all information entered here is included in the Methods section of the manuscript.**	N/A

**Format for specific study types**<br><br>**Human Subject Research (involving human participants and/or tissue)**<br>• Give the name of the institutional review board or ethics committee that approved the study<br>• Include the approval number and/or a statement indicating approval of this research<br>• Indicate the form of consent obtained (written/oral) or the reason that consent was not obtained (e.g. the data were analyzed anonymously)<br><br>**Animal Research (involving vertebrate animals, embryos or tissues)**<br>• Provide the name of the Institutional Animal Care and Use Committee (IACUC) or other relevant ethics board that reviewed the study protocol, and indicate whether they approved this research or granted a formal waiver of ethical approval<br>• Include an approval number if one was obtained<br>• If the study involved *non-human primates*, add *additional details* about animal welfare and steps taken to ameliorate suffering<br>• If anesthesia, euthanasia, or any kind of animal sacrifice is part of the study, include briefly which substances and/or methods were applied<br><br>**Field Research**<br><br>Include the following details if this study involves the collection of plant, animal, or other materials from a natural setting:<br>• Field permit number<br>• Name of the institution or relevant body that granted permission	
**Data Availability**<br><br>Authors are required to make all data underlying the findings described fully available, without restriction, and from the time of publication. PLOS allows rare exceptions to address legal and ethical concerns. See the PLOS Data Policy and FAQ for detailed information.	Yes - all data are fully available without restriction

A Data Availability Statement describing where the data can be found is required at submission. Your answers to this question constitute the Data Availability Statement and **will be published in the article**, if accepted.<br><br>**Important:** Stating 'data available on request from the author' is not sufficient. If your data are only available upon request, select 'No' for the first question and explain your exceptional situation in the text box.<br><br>Do the authors confirm that all data underlying the findings described in their manuscript are fully available without restriction?	
**Describe where the data may be found in full sentences. If you are copying our sample text, replace any instances of XXX with the appropriate details.**<br><br>• If the data are **held or will be held in a public repository**, include URLs, accession numbers or DOIs. If this information will only be available after acceptance, indicate this by ticking the box below. For example: *All XXX files are available from the XXX database (accession number(s) XXX, XXX.*).<br>• If the data are all contained **within the manuscript and/or Supporting Information files**, enter the following: *All relevant data are within the manuscript and its Supporting Information files.*<br>• If neither of these applies but you are able to provide **details of access elsewhere**, with or without limitations, please do so. For example:<br><br>*Data cannot be shared publicly because of [XXX]. Data are available from the XXX Institutional Data Access / Ethics Committee (contact via XXX) for researchers who meet the criteria for access to confidential data.*<br><br>*The data underlying the results presented in the study are available from (include the name of the third party*	All relevant data are within the manuscript and its Supporting Information files

and contact information or URL).<br>• This text is appropriate if the data are owned by a third party and authors do not have permission to share the data.<br><br>* typeset	
Additional data availability information:	Tick here if the URLs/accession numbers/DOIs will be available only after acceptance of the manuscript for publication so that we can ensure their inclusion before publication.; Tick here if your circumstances are not covered by the questions above and you need the journal's help to make your data available.

Revised Manuscript in PDF

Click here to access/download;Manuscript;Revised Manuscript Bayesian spatio-temporal.pdf

Bayesian spatio-temporal modeling of malaria risk in Rwanda

Muhammed Semakula [1,3], Francǫis Niragire [2], Christel Faes[1]

1 I-BioStat, Hasselt University, Hasselt, Belgium

2 College of Business and Economics, Department of Applied Statistics, University of Rwanda, Kigali, Rwanda

3 College of Business and Economics, Center of excellence in Data science, Bio-statistics, University of Rwanda, Kigali, Rwanda

Abstract

Every year, 435,000 people worldwide die from Malaria, mainly in Africa and Asia. However, malaria is a curable and preventable disease. Most countries are planning malaria elimination to meet sustainable development goal three, target 3.3, of ending malaria by 2030. Rwanda, through the malaria strategic plan 2012-2018 set a target to reduce malaria incidence by 42% from 2012-2018. Assessing the health policy and taking a decision using the incidence rate approach is becoming more challenging. We are proposing suitable statistical methods that handle spatial structure and uncertainty on the relative risk that is relevant to National Malaria Control Program.

We used spatio-temporal model to estimate the excess probability for decision making at a small area on evaluating reduction of incidence. SIR and BYM models were developed using Health facilities routine data from 2012-2018 in Rwanda. The fitted model was used to generate relative risk (RR) estimates comparing the risk with malaria risk in 2012, and to assess the probability of attaining the set target goal per area.

The results showed an overall increase in malaria in 2016 particularly. The 47.36% of all sectors in Rwanda failed to meet the target reduction from 2012 to 2018. Our approach of using excess probability method to evaluate attainment of target or identifying

threshold is a relevant statistical method, which will enable the Rwandan Government to sustaining malaria control and monitoring targeted interventions.

1 Introduction

Malaria remains a public health threat in developing countries, even though it is a preventable and curable disease. Every two minutes, the life of a child under age five is lost due to the disease [1]. There are a total of 435,000 deaths per year because of malaria, mainly in Africa and Asia [2]. Though some countries have achieved elimination of malaria, those with a high burden of disease have recorded an increase in malaria cases for the last decade. The Sub-Sahara Africa and India contributed eight percent to the global burden [2].

The WHO Global technical strategy for malaria (GTS) aims to eliminate malaria worldwide by 2030. WHO classified the countries and communities based on progress towards elimination (Control or Elimination). Malaria elimination is defined as the interruption of local transmission by reducing the rate of malaria cases to zero for a specific malaria parasite in a defined geographic area over particular time period. Malaria control is defined as a reduction of disease incidence, prevalence, morbidity or mortality to a locally acceptable level as a result of deliberate efforts. Most countries have placed malaria elimination on their health agenda by 2030, though fewer than 30 countries worldwide were certified malaria-free by WHO in the last 60 years [3] [8].

The Malaria elimination feasibility studies proved that it can be eliminated. Requires a strong health system that enables communities to access quality services, along with strong health information systems for tracking progress, effective surveillance, and public health response [3].

The Malaria strategic plan (MSP) 2012-2018 contained ambitious goals aimed at eliminating malaria death and reducing malaria morbidity to less than 5% test positivity rate by 2018 [5]. Contrary to expectation, the number of malaria cases has increased in Rwanda, with 10 times more cases in 2017 compared to 2011. The increase in malaria cases is often associated with the direct and indirect influence of climate change [6]. The 2016 mid-term review report (MTR) of MSP concluded that it is unlikely that will Rwanda to meet pre-elimination objectives and recommended that the applicability and

implementation of pre-elimination should not be reviewed in line with WHO Guidelines. The MTR acknowledged the performance level of health management information system (HMIS) [4] [5].

The Rwanda health sector strategic plan (HSSP III) stated five key strategies for pre-elimination phases and five indicators to be tracked that included reducing malaria prevalence among women and under-five children, reducing malaria incidence from 26/1000 in 2011 to 20/1000 in 2015 and 15/1000 in 2018, keep slide positivity below 5%, increasing number children under five sleeping in Long-Lasting Insecticidal Net(LLIN) to 82% in 2018 from 15% in 2011, reduce malaria proportional morbidity from 4 to 3 in 2018 and finally increasing percentage of Households with at least 1 LLIN installed from 82% to above 85% [7].

The elimination of malaria requires a strengthened surveillance system that enables early detection of all malaria infections and rapid effective response. The World Health Organization and Global Fund promote the use of a health information system. Most developing countries adopted District Health Information Software (DHIS) [9]. The DHIS is a free and open source platform for the management of routine health information with a primary focus on producing health statistics [10]. Rwanda's health system uses DHIS for data recording, reporting, and analysis. The statistical analysis offered by those tools is basic descriptive statistics and visualization. For the epidemiological surveillance of malaria, HMIS enables aggregation data in one platform from all health facilities in Rwanda. Those data are used for further statistical analysis to inform evidence based strategies to control malaria. The Rwanda Malaria control program uses WHO recommended operational methods to detect epidemic threshold. The method is to compare constant case count with mean $\pm$ 2 SD (standard deviation) or median + upper third quintile of previous years series data [11]. The incidence maps that serve for decision making rely on a fixed cut off to determine a high or low incidence rate. However, none of those estimation methods take into consideration the spatial uncertainty or account for the population at risk. Nevertheless, those methods are sensitive to outliers and unlikely to detect malaria patterns in low transmission areas [12]. Those approaches can help to visualize the overall dispersion around prevalence or incidence estimates but not any information linked to the uncertainty of exceedance or incidence threshold [14].

Currently, there is an increase in use of model-based approaches with data from

surveys as suggested by authors of the feasibility of the malaria elimination phase [15]. The surveys are often inadequately powered to detect very low levels of heterogeneous transmission and those surveys are performed periodically, most often every five years. In contrast, routinely collected clinical data are timely and local. Few studies have combined model-based approaches, routinely collected clinical data, and population census data to informal national malaria elimination efforts.

A model-based approach to study geospatial malaria trends is advantageous to identify risk factors in the general population and enable anticipated evidence based-decisions. The statistical models allow the inclusion of a variety of features that capture the variation of disease risk [16]. In this paper, s[illegible]ial disease mapping techniques will be used to investigate the geographical variation of malaria risk. We use routinely collected malaria data from health facilities in each sector of Rwanda to illustrate a formal assessment of pre-specified target goals, which can be used for decision making at a small geographical scale on evaluating reduction of incidence targets toward malaria pre-elimination phase. Understanding the disparities in broad areas, while useful, is unlikely to accurately reflect the heterogeneity in outcomes at the local level [19]. Malaria elimination efforts can benefit greatly by quantifying variation across population groups and small geographical areas. An understanding of the geographic patterns of malaria enables health decision making by health services agencies both in government, as well as non-governmental organizations for policy development, targeted interventions and adequately allocate resources at the area of greater need.

2 Materials and methods

2.1 Data source

We used malaria cases data from the Rwanda health information system (HMIS) reported from January 1, 2012 through December 31,2018. Over 95% of malaria cases reported through HMIS are laboratory confirmed in Rwanda [13]. The number of malaria cases are available at the level of the health centre and disaggregated by gender and age groups. Rwanda's health system is organized through five hierarchical levels: referral hospitals provide the highest levels of specialty care, followed by district hospitals and

health centers at sector level. The remaining two lower levels are community-based health services including health post and community health workers. Rwanda has 416 administration sectors and each has at least one health centre. For this analysis, we analyzed malaria cases at sector level. 12 % of cases were under five and not desegregated by sex thus were excluded in the analysis.

Population data were available from census year 2012. For population estimates over the remaining follow-up period, we used projections made based on 2012 census. Population data were downloaded on the following link `www.statistics.gov.rw/datasource/42`

2.2 SIR

We adapt the traditional approach of calculating the Standardized Incidence Ratio (SIR) in each area i $(i = 1, ..., n)$ and year t $(t = 2012, 2013, ..., 2018)$, correcting for the age, and gender- demographic structure in an area. We will use the SIR as a tool to investigate the change in malaria risk at time t as compared to a certain reference year, in our case, the year 2012. As result, we define the SIR as the ratio of the number of observed cases y_{it} to the number of expected cases E_{it} in the ith area at time t:

$$SIR_{it} = \frac{y_{it}}{E_{it}}, \tag{1}$$

with the expected number of cases calculated as

$$E_{it} = \sum_{j=1}^{J} N_{ijt} r_j \tag{2}$$

the r_j is the reference rates in age and gender-group j and N_{ijt} is the population in the area i, age-gender group j and time t:

$$r_j = \frac{y_j^{2012}}{N_j^{2012}} \tag{3}$$

where y_j^{2012} are the cases observed in age/gender group j in Rwanda in 2012, and N_j^{2012} is the census population for 2012 in Rwanda in the corresponding age/gender group.

To evaluate progress towards the reduction of malaria incidence set by Malaria

strategic plan 2012-2018, the reference rate is based on the malaria incidence in year 2012. This will enable comparison of malaria rates with subsequent years. The expected counts therefore represent the total number of Malaria cases that one would expect if the population in area i contracted the disease at the same rate as in 2012.

2.3 Model specification

As SIR uses information only from within an area, it might give uncertain estimates for small areas. Classical methods do not take into account the spatial dependence among the areas. Therefore, we use Bayesian disease mapping approaches that take into account the spatial dependence amongst neighboring areas.

A Bayesian disease mapping model consists of three components: the data model (i.e. the distribution of the data given the parameters), the process model (i.e. a description of underlying spatial trend) and the parameter model (i.e. the prior distribution of the parameters to be estimated) [21]. The data model is given by

$$Y_{it} \quad \sim \quad \text{Poisson}(E_{it}\theta_{it}), \tag{4}$$

where a Poisson distribution is appropriate since disease data are counts of number of cases and are non-negative. It is assumed that the mean is a product of the expected count E_{it} and the relative risk θ_{it}.

The process model describes the underlying structure of the relative risks. We used the spatio-temporal extension of the spatial Besag-York-Mollie (BYM) model, which is the CAR convolution model with two random effects, one spatially-structured area-specific random effect and one unstructured area-specific random effect [23] [20]

$$log(\theta_i) = \alpha + u_i + \upsilon_i + \gamma_t + \psi_t + \delta_{it} \tag{5}$$

where, u_i is the spatially-structured area-specific random effect which allows for smoothing amongst adjacent areas, namely [23]

$$u_i|u_j \sim N\left(\bar{\mu}_{\delta_i}, \frac{\sigma_u^2}{n_{\delta_i}}\right)$$

with δ_i and n_{δ_i} respectively, the set of neighbours and number of neighbours for a specific area i. The unstructured component υ_i is modeled using as a Gaussian process

$$\upsilon_i \sim N(0, \sigma_\upsilon^2),$$

and allows for extra heterogeneity in the counts due to unobserved (and spatially unstructured) risk factors. The γ_t term represents the temporally structured effect, modeled dynamically using random walk of order 2 (RW of order 2) and defined as

$$\gamma_t|\gamma_{t-1}, \gamma_{t-2} \sim N\left(2\gamma_{t-1} + \gamma_{t-2}, \sigma^2\right)$$

. The term ψ_t is specified by means of Gaussian exchangeable prior, defined as $\psi_t \sim N(0, \frac{1}{\tau_\psi})$. In order to allow for interaction between space and time, which explain differences in the time trend of malaria risk for different areas, the parameter δ_{it} follow a Gaussian Distribution with a precision matrix given by $\tau_\delta \mathbf{R}_\delta$, where τ_δ is unknown scalar, while $\mathbf{R}_\delta$ is the structure matrix, identifying the type of temporal and/ or spatial dependence between the elements of δ. For the interaction, we fitted models that consider four different types of interactions (Table 1), as presented in literature [24]. The best model was chosen basing deviance information criterion (DIC) [21].

Table 1. Interaction types:Parameter interacting and rank of $\mathbf{R}_\delta$

Type of interaction	structure matrix	Rank
Type I interaction	$\mathbf{R}_\delta = \mathbf{R}_\upsilon \bigotimes \mathbf{R}_\psi = I \bigotimes \mathbf{I} = \mathbf{I}$	nT
Type II interaction	$\mathbf{R}_\delta = \mathbf{R}_\upsilon \bigotimes \mathbf{R}_\gamma$	$n(T-2)$ for RW2
Type III interaction	$\mathbf{R}_\delta = \mathbf{R}_\psi \bigotimes \mathbf{R}_u$	$(n-1)T$
Type IV interaction	$\mathbf{R}_\delta = \mathbf{R}_u \bigotimes \mathbf{R}_\gamma$	$(n-1)(T-2)$ for RW2

The type I assumes that the two unstructured effect υ_i and ψ_t interact. Type II combines the structured temporal main effect γ_t and the unstructured spatial effect υ_i. Type III combines the unstructured temporal ψ_t and spatially structured main effect u_i. Finally, type IV is the most complex type of interaction, it assumes the spatially and temporally structured effects u_i and γ_t. We assigned a gamma distribution with shape equal 0.5 and rate equal to 0.00149 following the approach of Fong et al.(2010) [22]. For the remaining parameters, we assigned prior distributions to scaled precision matrix

parameters based on their marginal standard deviations on its diagonal following methods proposed by Sorby and Rue (2013) [18].

In order to investigate whether or not a reduction of malaria was observed compared to overall incidence rate in 2012, we make use of excess probability. The probability that the malaria risk has decreased by c% is calculated as the posterior probability $P(\theta_{it} < (100 - c)\%)$. If $|P|$ is large, the set goal is likely reached in that area, while if $|P|$ small, it is very likely not been reached.

2.4 Estimation methods

We used Integrated Nested Laplace approximation (INLA) for estimation. The INLA is a deterministic algorithm for Bayesian inference and is designed for latent Gaussian models and spatial models. Bayesian estimation using the INLA methodology takes much less time as compared to estimation using Markov Chain Monte Carlo Methods (MCMC). [17]

We performed a sensitivity analysis on variety of model formulations for the latent level due to inherent issues that come with each formulation. It is well known from literature that in BYM model, the spatially structured component cannot be seen independently from unstructured component. As an alternative model BYM2 improves parameter the control on those parameters, allowing the parameter to be seen independently from each other [18]. We fitted both models (BYM and BYM2) using the same priors. Results from both models were similar.

3 Results

The results are presented into two parts. The first part provides summary descriptive statistics of malaria cases and estimates from the fitted spatio-temporal model. The second part presents evaluation of Rwanda's malaria policy on the reduction of incidence using the excess probability approach. We introduced formal friendly interpretation and classification based on the excess probability approach for decision making during the malaria pre-elimination phase.

3.1 Malaria cases in Rwanda 2012-2018

Rwanda has experienced an increase in malaria cases from 398,287 cases per year in 2012 to 2,956,337 cases in 2016. However, in the last two years 2017 and 2018 the cases reduced to 1,978,450 and 1,725,522 respectively. Figure 1 shows the trend as overall and desegregated by age groups and sex. The year 2015 and 2016 recorded the highest number of cases, in all age/gender groups.

Fig 1. Malaria cases over time by sex

3.2 Malaria relative risk in Rwanda 2012-2018: BYM

We have fitted spatio-temporal models for seven years period, taking into account both structured and unstructured random effects (BYM and BYM2 models) as it provides a compromise between spatial correlation and extra heterogeneity over time. Since the results of those models are similar, we present BYM model fitted with type II interaction based on DIC 2.

Table 2. Comparison of models basing on DIC

Model	D	pD	DIC	DICc	WAIC
model.ST1	2848807	284.9672	2849092	2849422	2300753
mod.intI	640735.1	5251.247	645986.3	657421.2	941846.9
mod.intII	40046.5	14499.43	54545.93	91864.38	70889.18
mod.intIII	41886.02	16217.6	58103.63	97675.84	76402.72
mod.intIV	6.674699e+106	6.674699e+106	1.33494e+107	6.674699e+106	1.85816e+2[illegible]

Those models provide the estimates at the smallest available geographical scale, that might be an added value to drive oriented and targeted interventions to control malaria in Rwanda.

Fig 2. Posterior temporal trend effect for Malaria Relative Risk in Rwanda: $\exp(\phi_t + \gamma_t)$ with 95% Credible Interval

Estimates of variances due to random effects are presented in Table 3, the contribution of variance can be summarized, showing that about 50% is explained by a spatial component, and 50% by unstructured component. This is also visible in Figure 3, which presents estimated relative risks for each year, compared with the overall incidence rate year in 2012.

Table 3. Posterior mean and 95% Credibility interval for fixed effect of α

**Year**	**Parameter**	**Estimate**	**SD**	**LL**	**UL**
2012	σ_u^2	0.2703	0.0877	0.1414	0.4822
	σ_v^2	0.2407	0.0278	0.19	0.2991
$Frac_{spatial}$	$Var_u/(Var_u + \sigma_v^2)$	52%			
2013	σ_u^2	0.2696	0.0822	0.1454	0.4657
	σ_v^2	0.2668	0.0309	0.2107	0.332
$Frac_{spatial}$	$Var_u/(Var_u + \sigma_v^2)$	49%			
2014	σ_u^2	0.2942	0.0889	0.159	0.5059
	σ_v^2	0.2775	0.0307	0.2216	0.3421
$Frac_{spatial}$	$Var_u/(Var_u + \sigma_v^2)$	50.5%			
2015	σ_u^2	0.3981	0.1455	0.1925	0.7558
	σ_v^2	0.2928	0.0318	0.2342	0.3594
$Frac_{spatial}$	$Var_u/(Var_u + \sigma_v^2)$	56%			
2016	σ_u^2	0.6749	0.2602	0.3131	1.3203
	σ_v^2	0.3877	0.0392	0.3153	0.4691
$Frac_{spatial}$	$Var_u/(Var_u + \sigma_v^2)$	62%			
2017	σ_u^2	0.4947	0.1602	0.2576	0.8805
	σ_v^2	0.4135	0.0442	0.3326	0.5059
$Frac_{spatial}$	$Var_u/(Var_u + \sigma_v^2)$	53%			
2018	σ_u^2	0.3466	0.1016	0.192	0.5879
	σ_v^2	0.4846	0.0615	0.3735	0.6147
$Frac_{spatial}$	$Var_u/(Var_u + \sigma_v^2)$	41%			
2012-2018	$Frac_{spatial}$				
	$Var_u/(Var_u + \sigma_v^2)$	52%			

SD:Standard Deviation, LL: Lower Level, UL: Upper Level

Figure 2 shows an increasing trend effect for malaria relative risk in Rwanda with 95% Credible Interval over years.

In general, spatio-temporal contribution to geographic variability are important, as there is a tendency to see low relative risks in the North-West of Rwanda, and high relative risk in the East and in the South of Rwanda. We observe also a large amount of heterogeneity amongst areas, as some of the areas with high relative risk for Malaria are surrounded by areas with low risk (and vice versa). Table 4 shows the number of sectors

Fig 3. Malaria Relative Risk from year 2012 to 2018

with RR's within specific intervals.

In 2012, 73.8% (307) of all sectors (416) had RR < 1,thus with a lower than average disease rate, while 18.03% (75) of the sectors RR were above one but below 4, and 5.53% (23) above 4 but below 10. Eleven sectors RR was above ten, including four sectors with a relative risk greater than 15. Those four sectors were in the City of Kigali, with the highest RR observed in Gasabo District Gikomero sector (RR= 19.6, 95% CI = 19.13, 20.05). The two other sectors were in the Southern province Kigoma sector in Nyanza (RR= 19.7, 95% CI= 19.23, 20.25) and Gikonko in Gisagara District with a RR 16.8, 95% CI= 16.42, 17.20). The last one in Eastern province, Nyagatare District, was Nyagatare sector with a (RR= 15.75, 95% CI = 15.51, 16.01). This indicates that the malaria cases are concentrated in few areas, while the disease rate is low in most sectors.

In 2013, there is an increase in the number of sectors with RR ranging between one and four. In fact, the category of (1,4) increased to 22.12% as compared to 2012. In 2014, 36 (8.65%) sectors had RR > 4. For the year 2015, 39.9% sectors have higher RR > 1, and 5.3% of sectors had a RR > 4. In 2016, 40.87% of all the sectors had RR > 1 and 6.97% of sectors had RR > 4. In 2017, 37.98% of sectors had a RR > 1 and 9.13 of sectors had a RR > 4. Similar to previous year, in 2018 37.74% of sectors had RR > 1 and 7.69% of sectors had RR > 4. In conclusion, compared to the overall risk in the year 2012, the risk has increased in later years. In addition, the number of sectors with lower than average risk in the year 2012 has decreased over time.

Table 4. Malaria RR per year as compared to the year 2012

**Year**	**Malaria RR 2012:2018**				
	**[0,1[**	**[1,4[**	**[4,10[**	**[10,15[**	**[15,24[**
2012	307(73.80%)	75(18.03%)	23(5.53%)	7(1.68%)	4(0.96%)
2013	290(69.71%)	92(22.12%)	25(6.01%)	7(1.68%)	2(0.48%)
2014	278(66.83%)	102(24.52%)	30(7.21%)	3(0.72%)	3(0.72%)
2015	250(60.10%)	144(34.62%)	18(4.33%)	3(0.72%)	1(0.24%)
2016	246(59.13%)	141(33.89%)	27(6.49%)	2(0.48%)	0(0%)
2017	258(62.02%)	120(28.85%)	36 (8.65%)	2(0.48%)	0(0%)
2018	259(62.26%)	125(30.05%)	26(6.25%)	5(1.20%)	1(0.24%)

Fig 4. The Area-specific probabilities of not reaching the target goal of 2015 (reduction of 20% as compared to 2012)

Fig 5. The area-specific probability of not reaching the target goal of 2018 (reduction of 42% as compared to 2012)

3.3 Assessment of Malaria policy to reduce incidence in Rwanda

Rwanda Malaria's strategic plan 2012-2018 [5] aimed to reduce malaria incidence by 20 % in 2015 and 42% in 2018. Here results are showing explicitly the probability taking into account spatial uncertainty as it provides local details of the spatial variation of the risk. Figures 4 and 5 present the area-specific probabilities not reaching the target goals. Areas colored red have a high probability (above 80%) of not reaching the target goal, while areas in yellow have high probability (above 80%) of reaching the target goal. For areas in orange, we are uncertain about whether or not the sectors succeeded in achieving the target goals.

At the baseline year 2012, 29.33% (122) and 33.65% (140) of sectors had a high probability (> 0.8) to have smaller than average risk (< 0.58 and < 0.80, respectively). The number of sectors that failed to reach target of 20% reduction increased over the years. Similar to target of 42%, the number of sectors that failed to reach the target increased.

This is due to increased malaria incidence across all the sectors from 2012 to 2016. In 2017 and 2018, the incidence reduced, but not lower than in 2012. While an improvement towards reaching the target in some years is seen for some areas, the improvement did not persist over the entire follow-up period. After intervention of insecticide residual sp[illegible] (IRS) in 2015, 2016 and, 2017 the sectors of Nyagatare (East North) and Kirehe (East south) displayed reduction incidence. At the same time, we see that in the South-West, while targets were reached in the earlier years, these areas failed to sustain progress. Table 5 shows a summary of the number sectors that did not achieve the targets set out by Rwanda's malaria strategic plan with a certain probability.

Table 5. The sectors that did not achieve reducing the targets

Year	Target of reducing 20%				
	[0,0.2[	[0.2,0.4[	[0.4,0.6[	[0.6,0.8[	[0.8,1[
2012	289(69.47%)	1(0.24%)	4(0.96%)	0(0%)	122(29.33%)
2013	271(65.14%)	4(0.96%)	4(0.96%)	0(0%)	137(32.93%)
2014	258(62.02%)	1(0.24%)	1(0.24%)	4(0.96%)	152(36.54%)
2015	222(53.37%)	4 (0.96%)	0(0%)	5(1.20%)	185(44.47%)
2016	218(52.40%)	3(0.72%)	0(0%)	2(0.48%)	193(46.39%)
2017	236(56.73%)	2 (0.48%)	2(0.48%)	3(0.72%)	175(41.59%)
2018	241(57.93%)	2(0.48%)	2(0.48%)	2(0.48%)	169(40.62%)
	**Target of reducing 42% by 2018**				
2012	273(65.62%)	1(0.24%)	1(0.24%)	1(0.24%)	140(33.65%)
2013	250(60.10%)	3 (0.72%)	2(0.48%)	2 (0.48%)	159(38.22%)
2014	235(56.49%)	3(0.72%)	6(1.44%)	4(0.96%)	168(40.38%)
2015	200(48.08%)	2 (0.48%)	0(0%)	0(0%)	214(51.44%)
2016	187(44.59%)	1(0.24%)	2(0.48%)	0(0%)	226(54.33%)
2017	203(48.80%)	6(1.44%)	4(0.96%)	3(0.72%)	200(48.08%)
2018	216(51.92%)	0(0%)	3(0.72%)	4(0.96%)	193(46.39%)

4 Discussion

Spatial data has increased substantially due to the advances in computational tools that allow collection and integration of diverse real-time data sources. This goes in hand with the development of less or complex innovative statistical models to deal with the spatial structure of data in hand [24]. Model-based statistical methods are advantageous in low resource settings for estimating disease risk at health decision-making units as well as properties of uncertainty for survey data [25]. In this paper, this is extended towards the estimation of the probability to reach certain target goals.

In the past, a concern of data quality hampered the use of health facility data as a source of population based statistics. Introduction of web-based information systems for health facility data and implementation of universal health policy improved the completeness and accuracy of data at local areas to the extent of providing accurate statistics. This was fueled by the intensive monitoring of sustainable development goals [26] [27]. The data from health facilities in Rwanda are of high quality, though successfully integrating these data into health policy and decision-making throughout the health system is an ongoing challenge. [28].

The spatial modeling analysis for Rwanda malaria data from health facilities suggested an overall increase in relative risk (RR) in almost all sectors of Rwanda from 2012 to 2016, with a slight decrease from 2017 and 2018. The number of sectors with RR > 1

has increased tremendously, in some sectors the RR was above 10. This implies that malaria incidence slightly increased over time in all sectors of Rwanda but the increase was not persistent over years.

The estimated probability of achieving target for malaria reduction showed that, almost half (47.36%) of all sectors failed to meet the target of reducing 42% of malaria incidence by 2018, with 80% or 90% certainty. Contrary to the expectation from the Malaria Strategic plan [5], malaria incidence increased in East, South, Central, and West-south of Rwanda. Those areas of Rwanda are known as high malaria risk zones [5]. This means that the malaria control program should concentrate effort on reducing transmission through preventive interventions such as indoor residual spraying (IRS) and bed-net distribution. In 2013, 2015, 2016 and, 2017 as figure 4 and 5 show a change in the east-north (Nyagatare) and east-south (Kirehe); the reduction might be due to the IRS intervention that occurred in the same period in those Districts. With 90% probability, 51.92% of sectors reduced malaria incidence as planned; however, those sectors belong in Northern provinces and West-North of Rwanda where malaria cases are often relatively low as compared to other parts of Rwanda. Despite those encouraging success, much work remains to achieve malaria reduction targets for the whole country. Implementing pre-elimination strategies in those sectors should be premature, instead the focus should be implementing malaria control strategies.

The results presented here are based only on spatial analysis of malaria cases from health facilities and population distribution, and the database had limited variables that could have been included in the analysis to explain increased relative risk and reason of failing to achieve the target of reducing incidence as planned. We limited our scope on statistical method to evaluate reduction of malaria incidence using an excess probability approach. This approach is relevant tool to assess a health policy and guide the decision makers. It can contribute to improve Malaria surveillance to ensure appropriate intervention in the right place and at the right time.

A disease like Malaria requires a strong surveillance system that enable a quick response to any changes in Malaria behaviour. Efficient algorithms that can be deployed in response to real-time data collection and make inferences would contribute fast response to potential public health threats. [16]

5 Conclusion

In summary, we recommend the approach of using spatio-temporal models and routinely collected facility-based malaria data to assess the malaria targets related to incidence rate and estimate malaria relative risk at the local area level. This approach enables us to generate maps that provide information about the probability and uncertainty of reaching the target goal, as well as providing information on the spatial contribution to Malaria. The proposed approach is not only limited to malaria data, but it can also be applied in other health care delivery.

This era of sustainable development goals (SDGs), especially SDG 3 and its target 3.3 of ending malaria by 2030, requires a tool like the one presented here for planning, monitoring, and evaluation. The excess probability can be applied to survey or routine data from health facilities. It efficiently uses routine data for permanently monitoring the changes in malaria transmission and evaluation progress towards national targets. Though survey data are important, provided that data quality are high, routinely collected data are collected more frequently and thus provide more timely assessments of health burden. Most of the surveys, take five years to get new evidence, an example of DHS (Demographic and Health survey) and often do not provide estimates at a local level.

Acknowledgments

The authors are grateful to Rwanda Biomedical Center, Malaria and other Parasitic Diseases division, for collaboration and discussions on Rwanda Malaria's strategic plan evaluation approaches. We are grateful for Rwanda malaria resourceful materials shared by Dr. Aimable Mbituyumuremyi, Malaria Division manager and technical support provided by Mr. Hamza Ndabateze, HMIS officer, to extract data from Rwanda health information system (HMIS).

References

1. WHO. World Malaria report 2015[Internet]. Geneva; 2015. Available from: https://www.who.int/malaria/publications/world-malaria-report-

2015/report/en/

2. WHO. World Malaria report 2018 [Internet]. Geneva; 2018. Available from: https://www.who.int/malaria/publications/world-malaria-report-2018/en/

3. World Health Organization, Global Malaria Programme. A framework for malaria elimination [Internet]. WHO Press, World Health Organization. 2017. 100p. Available from: http://apps.who.int/iris/bitstream/handle/10665/254761/9789241511988-eng.pdf?sequence=1

4. Ministry of Health (Rwanda). Rwanda Malaria Strategic Plan (2013-2018) Mid Term Review. Rwanda Biomedical Centre. 2016. Available from: http://www.moh.gov.rw/index.php?id=511

5. Rwanda Biomedical Center(RBC). Rwanda Malaria Strategic Plan 2012-2018. RBC:Kigali, Rwanda. 2012.

6. Tesi M. Africa initiative Discussion Papers Global Warming and Health: The Issue of Malaria in Eastern African's Highlands. Global health. 2011; (2).

7. Ministry of Health (Rwanda). Rwanda third health sector strategic plan (2012-2018). Ministry of Health. 2016. Available from: http://www.moh.gov.rw/index.php?id=511

8. Hemingway, J. et al. Tools and Strategies for Malaria Control and Elimination: What Do We Need to Achieve a Grand Convergence in Malaria? PLoS Biology, 2016. 14(3), pp.1–14

9. Dehnavieh, R., Haghdoost, A., Khosravi, A., Hoseinabadi, F., Rahimi, H., Poursheikhali, A., ... Aghamohamadi, S. The District Health Information System (DHIS2): A literature review and meta-synthesis of its strengths and operational challenges based on the experiences of 11 countries. Health Information Management Journal, 2019.48(2), 62–75. https://doi.org/10.1177/1833358318777713

10. Sahay, S., Sæbø, J. & Braa, J. Scaling of HIS in a global context: Same, same, but different. Information and Organization journal, 2013. 23(4), pp.294–323. Available at: http://dx.doi.org/10.1016/j.infoandorg.2013.08.002.

11. WHO,2018, Malaria surveillance, monitoring and evaluation: A reference manual

12. World Health Organization. Epidemiological approach for Malaria control. World Health Organization, 2015. 2nd edition Available at: https://apps.who.int/iris/handle/10665/96351

13. USAID. President's Malaria initiative Rwanda operational plan financial year 2019. Available: `https://www.pmi.gov/docs/default-source/default-document-library/malaria-operational-plans/fy19/fy-2019-rwanda-malaria-operational-plan.pdf?sfvrsn=3`. Accessed 2019 September 27

14. Emmanuel Giorgi et al. Using non-exceedance probabilities of policy-relavant malaria prevalence thresholds to identify areas of low transmission in Somalia Malaria Journal, 2018. 17, Article number: 88

15. Andrew J Tatem, David L Smith, Peter W Gething, Caroline W Kabaria, Robert W Snow, Simon I Hay. Ranking of elimination feasibility between malaria-endemic countries. Lancet 2010; 376: 1579–91

16. Lawson, A. & Lee, D. Bayesian Disease Mapping for Public Health. Handbook of statistics, 2017. volume 36, Pages 443-481. Available at: http://dx.doi.org/10.1016/bs.host.2017.05.001

17. Carroll, R. et al. Comparing INLA and OpenBUGS for hierarchical Poisson modeling in disease mapping. Spatial and Spatio-temporal Epidemiology, 2015. 14–15, pp.45–54 available at:doi: 10.1016/j.sste.2015.08.001

18. Andrea Riebler and Sigrunn H Sørbye and Daniel Simpson and Håvard Rue. An intuitive Bayesian spatial model for disease mapping that accounts for scaling. Statistical Methods in Medical Research, 2016. 25,4, pp.1145–1165 available at:doi: 10.1177/0962280216660421

19. Su Yun Kang,Susanna M. Cramb, Nicole M. White, Stephen J. Ball,Kerrie L. Mengersen,2016. Making the most spatial information in health: A tutorial in Bayesian Disease Mapping for areal Data Geospatial health Journal. 2016. 31;11(2):428 available at: DOI: 10.4081/gh.2016.428

20. Besag, J., & Green, P. Spatial Statistics and Bayesian Computation. Journal of the Royal Statistical Society. Series B (Methodological), 1993. 55(1), 25-37.

21. Lesaffre,E. and A. B. Lawson. Bayesian Biostatistics.Statistics in practice. UK:John Wiley & Sons. 2012 Available at:DOI:10.1002/9781119942412

22. Fong, Youyi and Rue, Håvard and Wakefield, Jon. Bayesian inference for generalized linear mixed models. Biostatistics. 2010 11, 3, 397-412 Available at:DOI:10.1093/biostatistics/kxp053

23. Besag, J., York, J., & Mollie (1991) Baysian image restoration with two applications in spatial statistics. Ann. Inst. Statist. Math.1991 43: 1. available at:https://doi.org/10.1007/BF00116466

24. Marta Blangiordo, & Michela Cameletti. Spatial and Spatio temporal Bayesian models with R-INLA. UK:John Wiley & Sons.2015 available at:DOI:10.1002/9781118950203

25. Robert Yankson, Evelyn Arthur Anto & Michael Give Chipeta. Geostatistical analysis and mapping of malaria risk in children under 5 using point-referenced prevalence data in Ghana. Malaria Journal. 2019. 18:67 available at:https://doi.org/10.1186/s12936-019-2709-y

26. Sabella Maina, Pepela Wanjala, David Soti,Hillary Kipruto, Benson Drotid & Ties Boermae. Using health-facility data to assess subnational coverage of maternal and child health indicators, Kenya. Bull World Health organ. 2017.1;95:683:694 available at:95(10):683-694. doi: 10.2471/BLT.17.194399

27. Marie Paul Nisingizwe, Hari S. Iyer, Modeste Gashayija, Lisa R. Hirschhorn, Cheryl Amoroso, Randy Wilson, Eric Rubyutsa, Eric Gaju, Paulin Basinga, Andrew Muhire, Agne's Binagwaho, Bethany Hedt-Gauthier. Toward utilization of data for program management and evaluation: Quality assessment of five years of health management information system data in Rwanda. Global Health Action. 2014.7 available at: ISSN:1654-9716. doi: 10.3402/gha.v7.25829

28. Karengera Innocent, Robert Anguyo DDM Onzima, Simon-Peter, Philip Govule. Quality and Use of Routine Healthcare Data in Selected Districts of Eastern

Province of Rwanda. international journal for public health research 2016.(2):5:13 available at:ISSN:2381-4837

Fig1A Revised Click here to access/download;Figure;Fig1A.tif

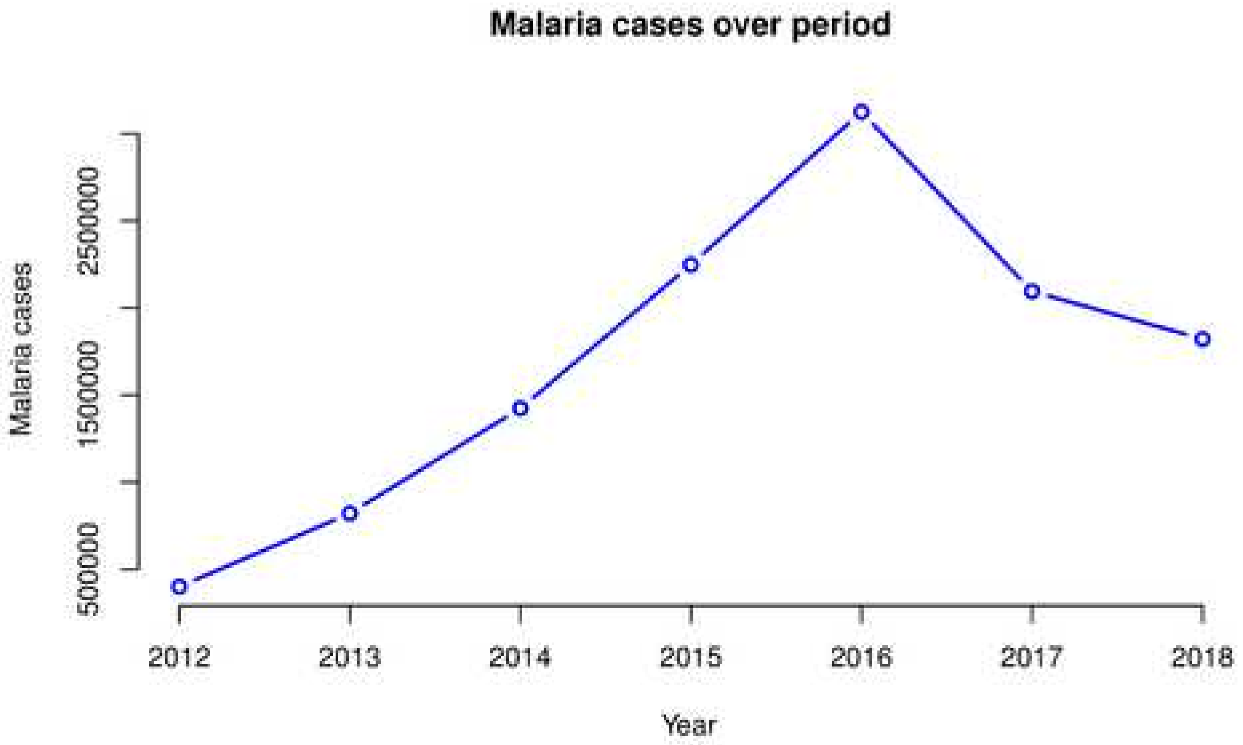

 Click here to access/download;Figure;Fig1B.tif

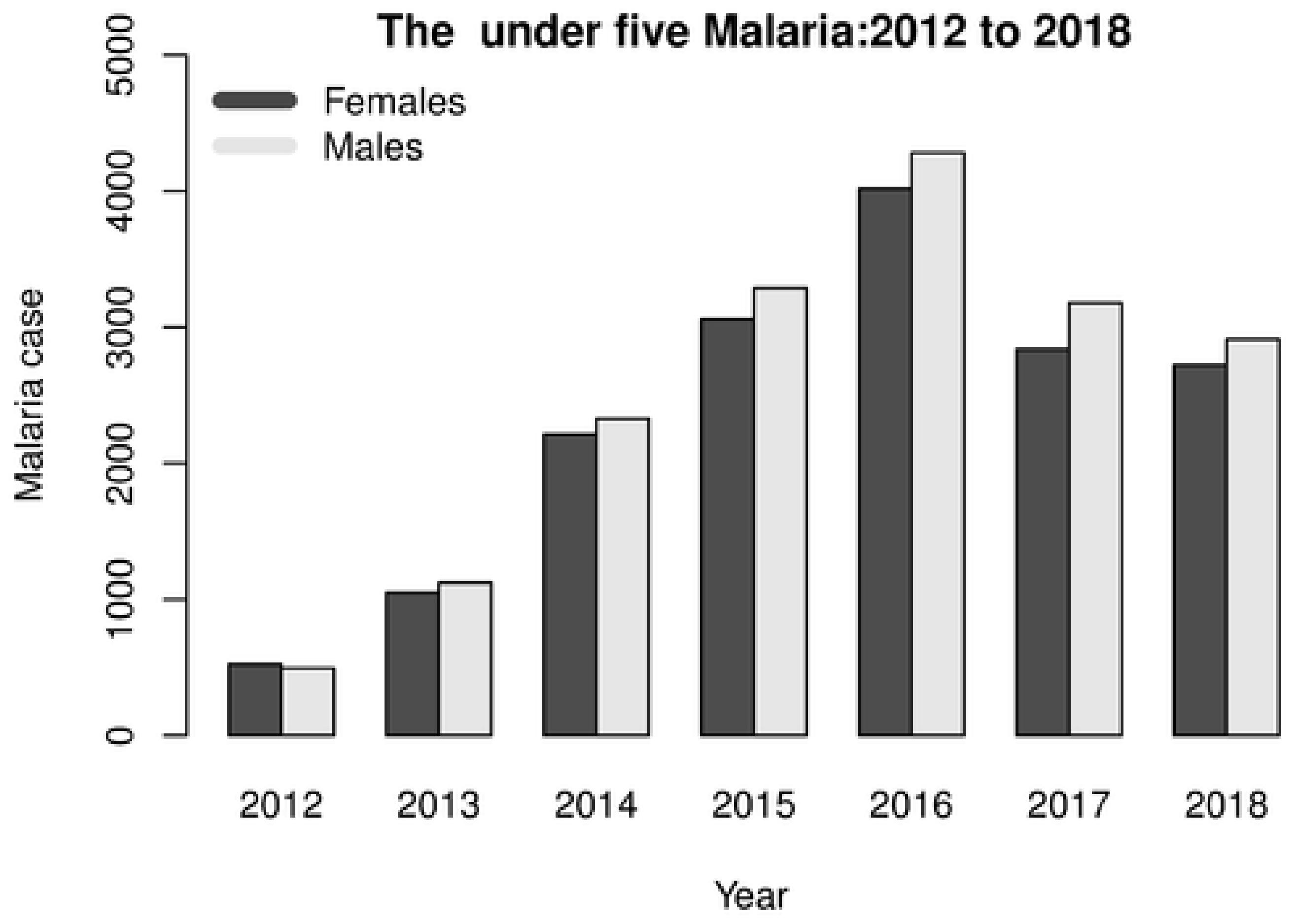

Fig1C Revised

Click here to access/download;Figure;Fig1C.tif

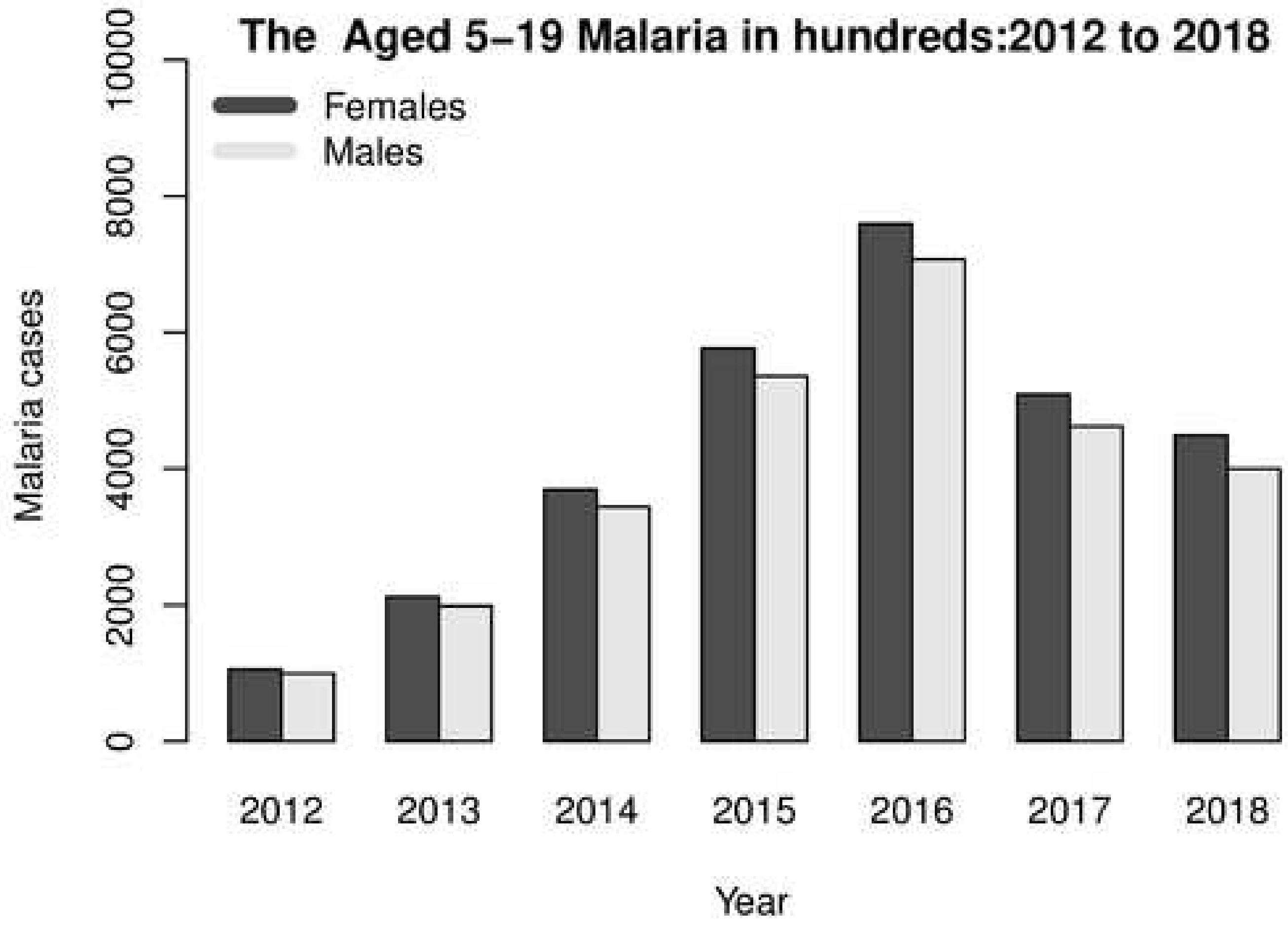

 Click here to access/download;Figure;Fig1D.tif

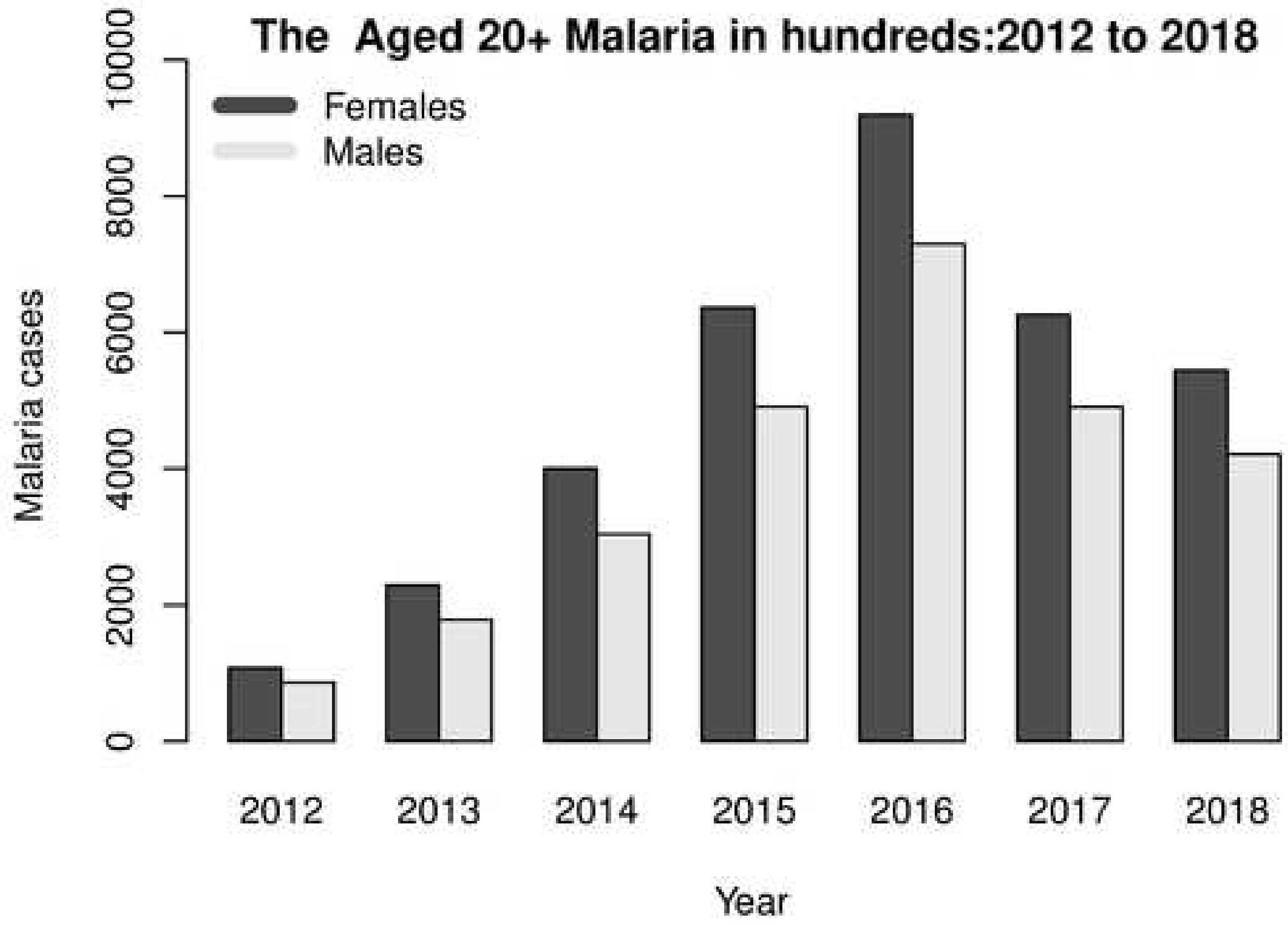

Click here to access/download;Figure;Fig2.tif

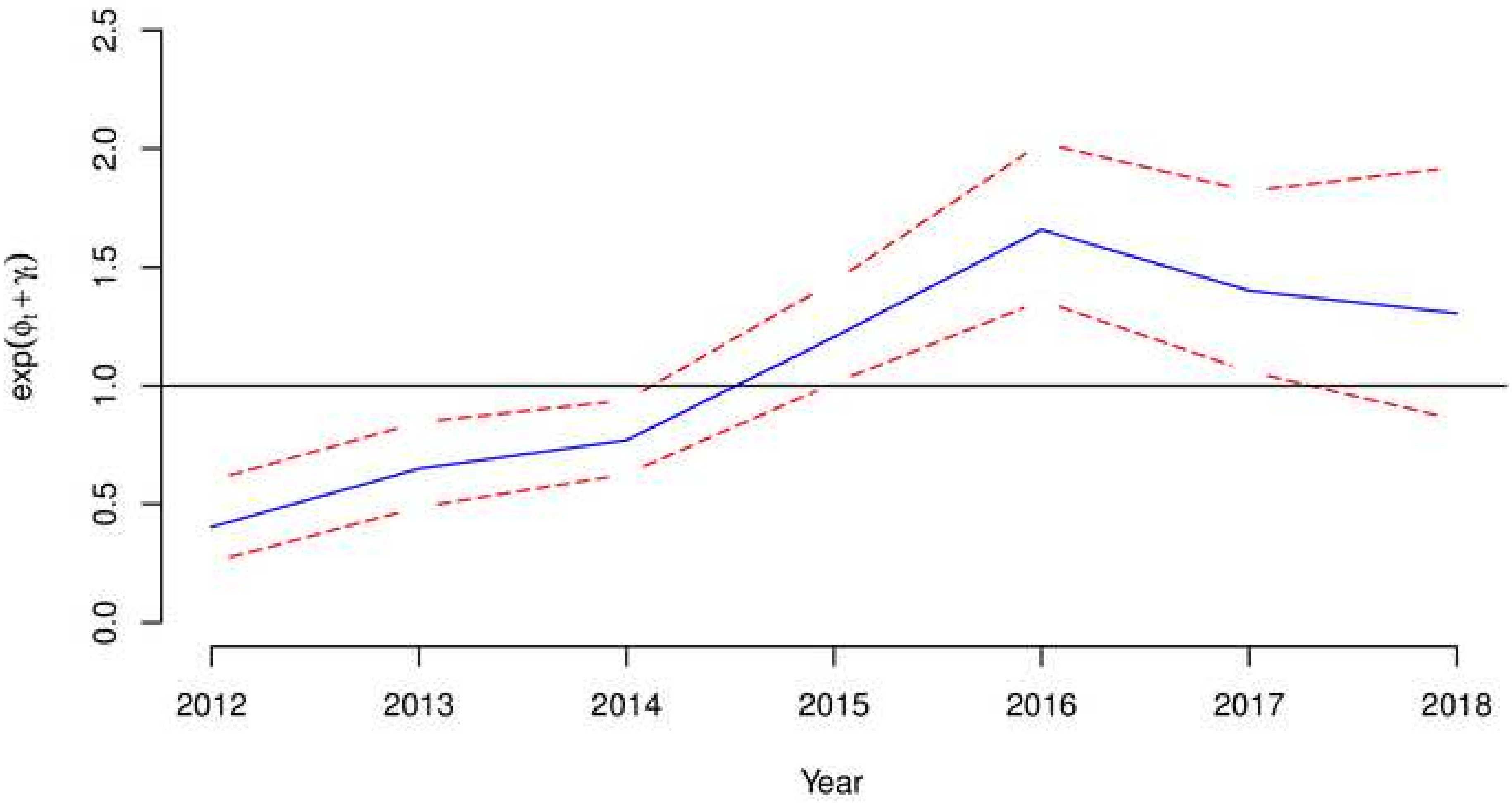

Fig3 Revised

Click here to access/download;Figure;Fig3.tif

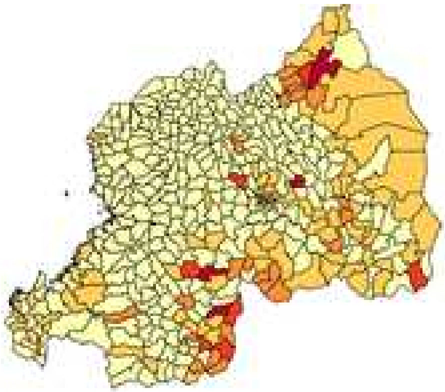

(a) RR in 2012

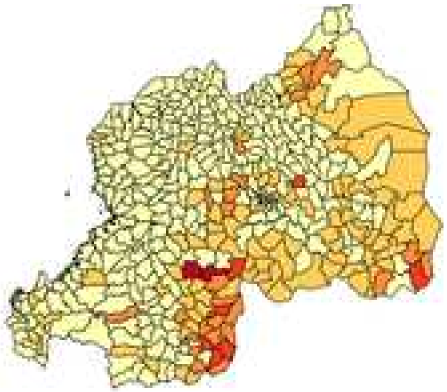

(b) RR in 2013

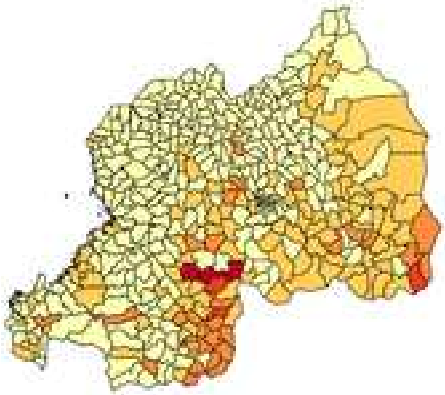

(c) RR in 2014

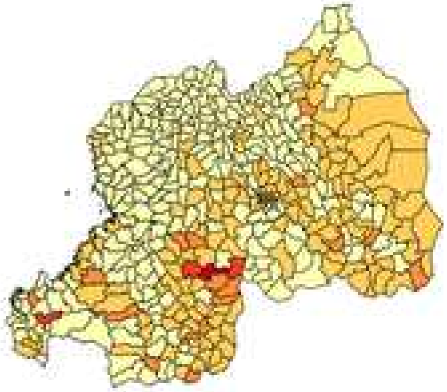

(d) RR in 2015

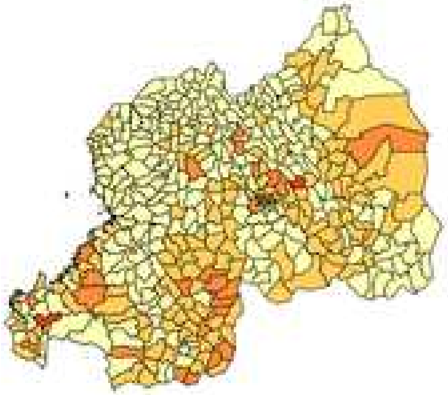

(e) RR in 2016

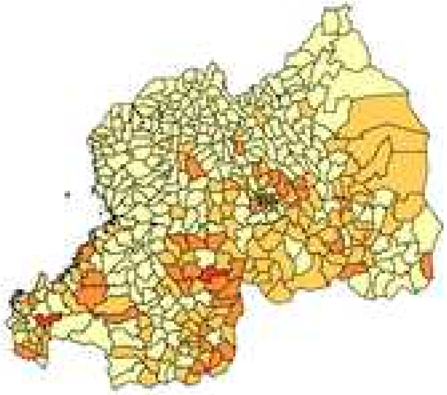

(f) RR in 2017

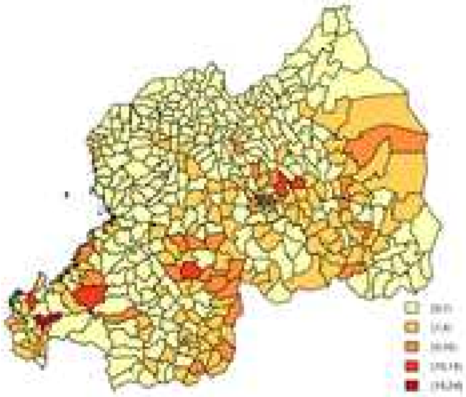

(g) RR in 2018

Fig4 Revised
Click here to access/download;Figure;Fig4.tif

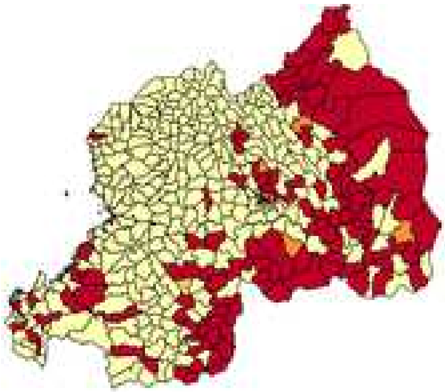

(a) 2012

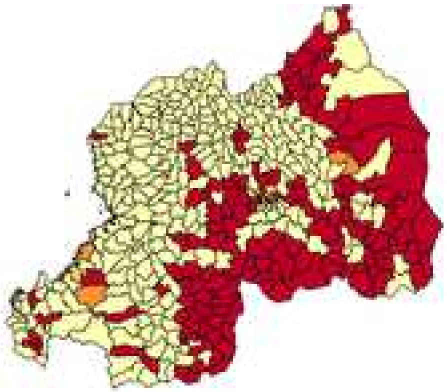

(b) 2013

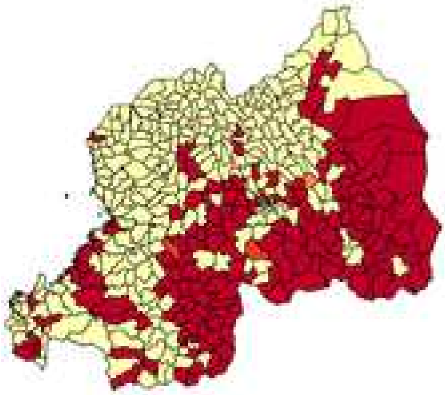

(c) 2014

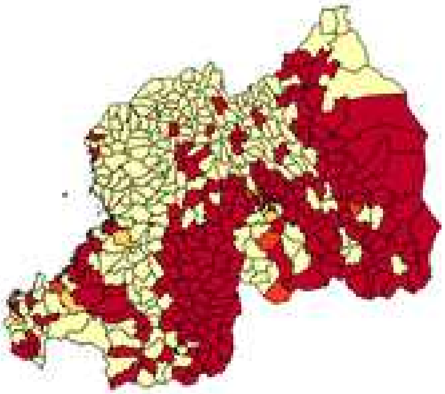

(d) 2015

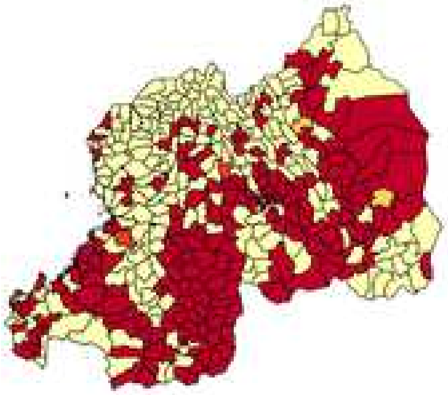

(e) 2016

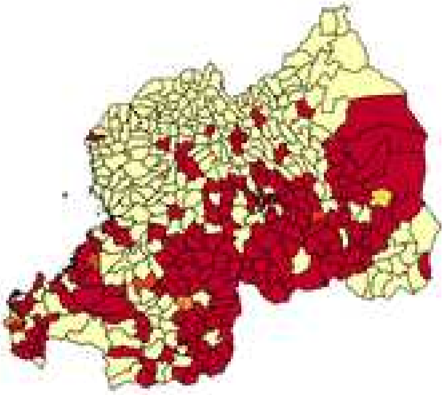

(f) 2017

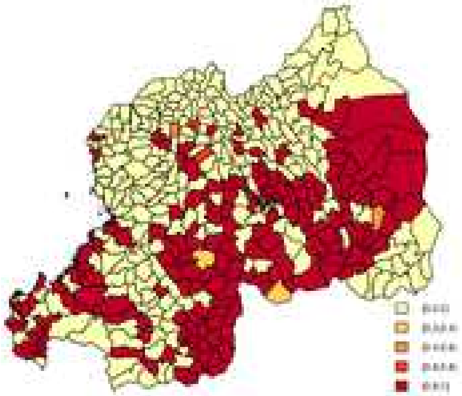

(g) 2018

Fig5 Revised

Click here to access/download;Figure;Fig5.tif

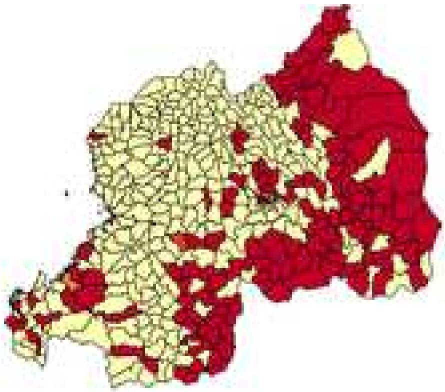

(a) 2012

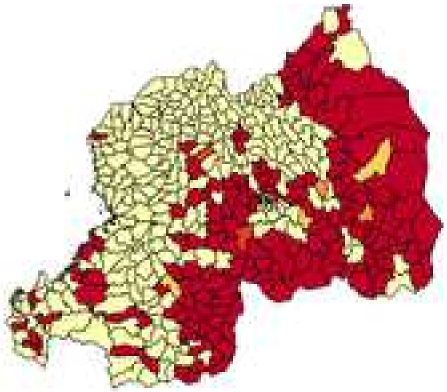

(b) 2013

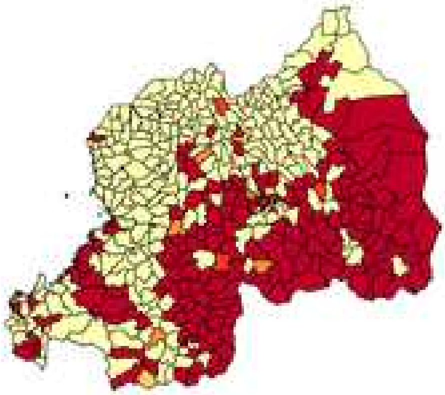

(c) 2014

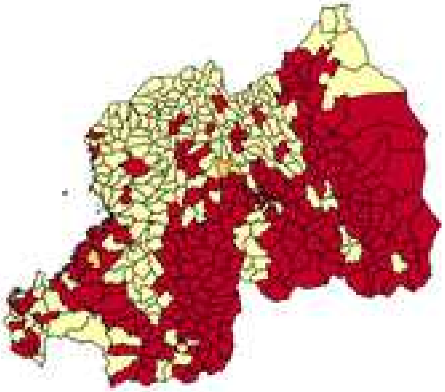

(d) 2015

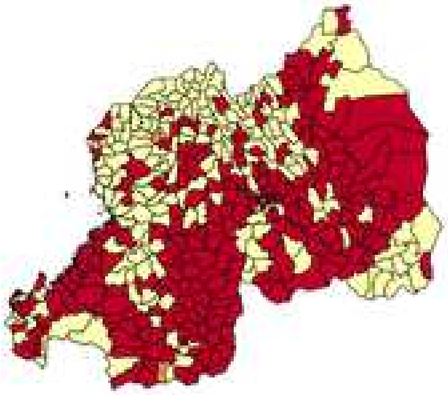

(e) 2016

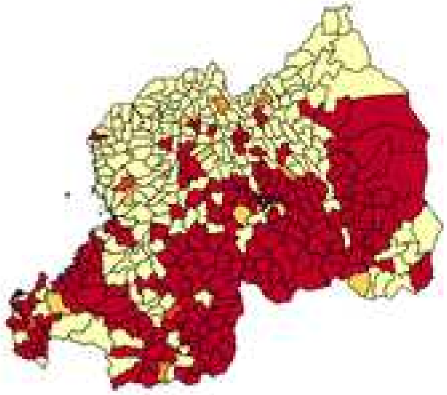

(f) 2017

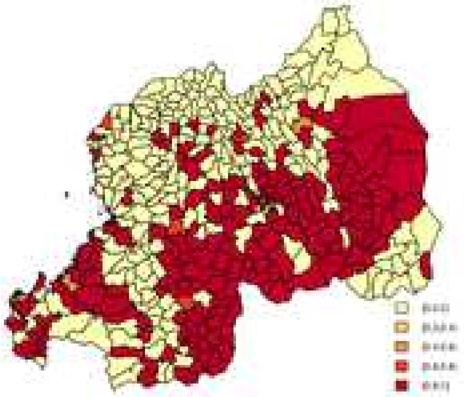

(g) 2018

Click here to access/download
Supporting Information
codes_Data.R

data

Click here to access/download
Supporting Information
3944de3f-aa07-448b-b206-71598066f9d6

Bayesian spatial spatio-temporal modeling of malaria risk in Rwanda

Muhammed Semakula [1,3], Francǫis Niragire [2], Christel Faes[1]

1 I-BioStat, Hasselt University, Hasselt, Belgium

2 College of Business and Economics, Department of Applied Statistics, University of Rwanda, Kigali, Rwanda

3 College of Business and Economics, Center of excellence in Data science, Bio-statistics, University of Rwanda, Kigali, Rwanda

Abstract

Every year, 435,000 people worldwide die from Malaria, mainly in Africa and Asia. However, malaria is a curable and preventable disease. Most countries are planning malaria elimination to meet sustainable development goal three, target 3.3, of ending malaria by 2030. Rwanda, through the malaria strategic plan 2012-2018 set a target to reduce malaria incidence by 42% from 2012-2018. Assessing the health policy and taking a decision using the incidence rate approach is becoming more challenging. We are proposing suitable statistical methods that handle spatial structure and uncertainty on the relative risk that is relevant to National Malaria Control Program.

We used spatial spatio-temporal model based methods to estimate the excess probability for decision making at a small area on evaluating reduction of incidence. SIR and BYM models were developed using Health facilities routine data from 2012-2018 in Rwanda. The fitted model for BYM was used to generate relative risk (RR) estimates comparing the risk with malaria risk in 2012, and to assess the probability of attaining the set target goal per area.

The results showed an overall increase in malaria in 2016 particularly.and The 47.36% of all sectors in Rwanda failed to meet the target reduction from 2012 to 2018.

Our approach of using excess probability method to evaluate attainment of target or identifying threshold is a relevant statistical method, which will enable the Rwandan Government to sustaining malaria control and monitoring targeted interventions.

1 Introduction

Malaria remains a public health threat in developing countries, even though it is a preventable and curable disease. Every two minutes, the life of a child under age five is lost due to the disease [1]. There are a total of 435,000 deaths per year because of malaria, mainly in Africa and Asia [2]. Though some countries have achieved elimination of malaria, those with a high burden of disease have recorded an increase in malaria cases for the last decade. The Sub-Sahara Africa and India contributed eight percent to the global burden [2].

The WHO Global technical strategy for malaria (GTS) aims to eliminate malaria worldwide by 2030. WHO classified the countries and communities based on progress towards elimination (Control or Elimination). Malaria elimination is defined as the interruption of local transmission by reducing the rate of malaria cases to zero for a specific malaria parasite in a defined geographic area over particular time period. Malaria control is defined as a reduction of disease incidence, prevalence, morbidity or mortality to a locally acceptable level as a result of deliberate efforts. Most countries have placed malaria elimination on their health agenda by 2030, though fewer than 30 countries worldwide were certified malaria-free by WHO in the last 60 years [3] [8].

The Malaria elimination feasibility studies proved that it can be eliminated. Requires a strong health system that enables communities to access quality services, along with strong health information systems for tracking progress, effective surveillance, and public health response [3].

The Malaria strategic plan (MSP) 2012-2018 contained ambitious goals aimed at eliminating malaria death and reducing malaria morbidity to less than 5% test positivity rate by 2018 [5]. Contrary to expectation, the number of malaria cases has increased in Rwanda, with 10 times more cases in 2017 compared to 2011. The increase in malaria cases is often associated with the direct and indirect influence of climate change [6]. The 2016 mid-term review report (MTR) of MSP concluded that it is unlikely that will

Rwanda to meet pre-elimination objectives and recommended that the applicability and implementation of pre-elimination should not be reviewed in line with WHO Guidelines. The MTR acknowledged the performance level of health management information system (HMIS) [4] [5].

The Rwanda health sector strategic plan (HSSP III) stated five key strategies for pre-elimination phases and five indicators to be tracked that included reducing malaria prevalence among women and under-five children, reducing malaria incidence from 26/1000 in 2011 to 20/1000 in 2015 and 15/1000 in 2018, keep slide positivity below 5%, increasing number children under five sleeping in Long-Lasting Insecticidal Net(LLIN) to 82% in 2018 from 15% in 2011, reduce malaria proportional morbidity from 4 to 3 in 2018 and finally increasing ~~%~~ percentage of Households with at least 1 LLIN installed from 82% to above 85% [7].

The elimination of malaria requires a strengthened surveillance system that enables early detection of all malaria infections and rapid effective response. The World Health Organization and Global Fund promote the use of a health information system. Most developing countries adopted District Health Information Software (DHIS) [9]. The DHIS is a free and open source platform for the management of routine health information with a primary focus on producing health statistics [10]. Rwanda's health system uses DHIS for data recording, reporting, and analysis. The statistical analysis offered by those tools is basic descriptive statistics and visualization. For the epidemiiological surveillance of malaria,~~HMIS enables to avail of data from all the health facilities in Rwanda for analysis and use for evidence-based strategies~~ HMIS enables aggregation data in one platform from all health facilities in Rwanda. Those data are used for further statistical analysis to inform evidence based strategies to control malaria. The Rwanda Malaria control program uses WHO recommended operational methods to detect epidemic threshold. The method is to compare constant case count with mean $\pm$ 2 SD (standard deviation) or median + upper third quintile of previous years series data [11]. The incidence maps that serve for decision making rely on a fixed cut off to determine a high or low incidence rate. However, none of those estimation methods take into consideration the spatial uncertainty or account for the population at risk. Nevertheless, those methods are sensitive to outliers and unlikely to detect malaria ~~patterns in low areas transmission~~ patterns in low transmission areas [12]. Those approaches can help

to visualize the overall dispersion around prevalence or incidence estimates but not any information linked to the uncertainty of exceedance or incidence threshold [14].

Currently, there is an increase in use of model-based approaches with data from surveys as suggested by authors of the feasibility of the malaria elimination phase [15]. The surveys are often inadequately powered to detect very low levels of heterogeneous transmission and those surveys are performed periodically, most often every five years. In contrast, routinely collected clinical data are timely and local. Few studies have combined model-based approaches, routinely collected clinical data, and population census data to informal national malaria elimination efforts.

A model-based approach to study geospatial malaria trends is advantageous to identify risk factors in the general population and enable anticipated evidence based-decisions. The statistical models allow the inclusion of a variety of features that capture the variation of disease risk [16]. In this paper, spatial disease mapping techniques will be used to investigate the geographical variation of malaria risk. We use routinely collected malaria data from health facilities in each sector of Rwanda to illustrate a formal assessment of pre-specified target goals, which can be used for decision making at a small geographical scale on evaluating reduction of incidence targets toward malaria pre-elimination phase. Understanding the disparities in broad areas, while useful, is unlikely to accurately reflect the heterogeneity in outcomes at the local level [19]. Malaria elimination efforts can benefit greatly by quantifying variation across population groups and small geographical areas. An understanding of the geographic patterns of malaria enables health decision making by health services agencies both in government, as well as non-governmental organizations for policy development, targeted interventions and adequately allocate resources at the area of greater need.

2 Materials and methods

2.1 Data source

We used malaria cases data from the Rwanda health information system (HMIS) reported from January 1, 2012 through December 31,2018. Over 95% of malaria cases reported through HMIS are laboratory confirmed in Rwanda [13]. The number of malaria cases

are available at the level of the health centre and disaggregated by gender and age groups. A data quality assessment was performed routinely on the data, resulting in fully completeness in the data. Rwanda's health system is organized through five hierarchical levels: referral hospitals provide the highest levels of specialty care, followed by district hospitals and health centers at sector level. The remaining two lower levels are community-based health services including health post and community health workers. Rwanda has 416 administration sectors and each has at least one health centre. For this analysis, we analyzed malaria cases at sector level.12 % of cases were under five and not desegregated by sex thus were excluded in the analysis ~~some cases of under five were not desegregated by sex thus were excluded in the analysis~~.

Population data were available from census year 2012. For population estimates over the remaining follow-up period, we used projections made based on 2012 census. Population data were downloaded on the following link `www.statistics.gov.rw/datasource/42`

2.2 SIR

We adapt the traditional approach of calculating the Standardized Incidence Ratio (SIR) in each area i $(i = 1, ..., n)$ and year t $(t = 2012, 2013, ..., 2018)$, correcting for the age, and gender- demographic structure in an area. We will use the SIR as a tool to investigate the change in malaria risk at time t as compared to a certain reference year, in our case, the year 2012. As result, we define the SIR as the ratio of the number of observed cases y_{it} to the number of expected cases E_{it} in the ith area at time t:

$$SIR_{it} = \frac{y_{it}}{E_{it}}, \quad (1)$$

with the expected number of cases calculated as

$$E_{it} = \sum_{j=1}^{J} N_{ijt} r_j \quad (2)$$

the r_j is the reference rates in age and gender-group j and N_{ijt} is the population in the area i, age-gender group j and time t:

$$r_j = \frac{y_j^{2012}}{N_j^{2012}} \tag{3}$$

where y_j^{2012} are the cases observed in age/gender group j in Rwanda in 2012, and N_j^{2012} is the census population for 2012 in Rwanda in the corresponding age/gender group.

To evaluate progress towards the reduction of malaria incidence set by Malaria strategic plan 2012-2018, the reference rate is based on the malaria incidence in year 2012. This will enable comparison of malaria rates with subsequent years. The expected counts therefore represent the total number of Malaria cases that one would expect if the population in area i contracted the disease at the same rate as in 2012.

2.3 Model specification

As SIR uses information only from within an area, it might give uncertain estimates for small areas. Classical methods do not take into account the spatial dependence among the areas. Therefore, we use Bayesian disease mapping approaches that take into account the spatial dependence amongst neighboring areas.

A Bayesian disease mapping model consists of three components: the data model (i.e. the distribution of the data given the parameters), the process model (i.e. a description of underlying spatial trend) and the parameter model (i.e. the prior distribution of the parameters to be estimated) [21]. The data model is given by

$$Y_{it} \sim \text{Poisson}(E_{it}\theta_{it}), \tag{4}$$

where a Poisson distribution is appropriate since disease data are counts of number of cases and are non-negative. It is assumed that the mean is a product of the expected count E_{it} and the relative risk θ_{it}.

The process model describes the underlying structure of the relative risks. We used the spatio-temporal extension of the spatial Besag-York-Mollie (BYM) model, which is the CAR convolution model with two random effects, one spatially-structured area-specific random effect and one unstructured area-specific random effect [23] [20]

$$log(\theta_i) = \alpha + u_i + \upsilon_i + \gamma_t + \psi_t + \delta_{it} \quad (5)$$

where, u_i is the spatially-structured area-specific random effect which allows for smoothing amongst adjacent areas, namely [23]

$$u_i | u_j \sim N\left(\bar{\mu}_{\delta_i}, \frac{\sigma_u^2}{n_{\delta_i}}\right)$$

with δ_i and n_{δ_i} respectively, the set of neighbours and number of neighbours for a specific area i. The unstructured component υ_i is modeled using as a Gaussian process

$$\upsilon_i \sim N(0, \sigma_\upsilon^2),$$

and allows for extra heterogeneity in the counts due to unobserved (and spatially unstructured) risk factors.The γ_t term represents the temporally structured effect, modeled dynamically using random walk of order 2 (RW of order 2) and defined as

$$\gamma_t | \gamma_{t-1}, \gamma_{t-2} \sim N\left(2\gamma_{t-1} + \gamma_{t-2}, \sigma^2\right)$$

. The term ψ_t is specified by means of Gaussian exchangeable prior, defined as $\psi_t \sim N(0, \frac{1}{\tau_\psi})$. In order to allow for interaction between space and time, which explain differences in the time trend of malaria risk for different areas, the parameter δ_{it} follow a Gaussian Distribution with a precision matrix given by $\tau_\delta \mathbf{R}_\delta$, where τ_δ is unknown scalar, while $\mathbf{R}_\delta$ is the structure matrix, identifying the type of temporal and/ or spatial dependence between the elements of δ. For the interaction, we fitted models that consider four different types of interactions (Table 1), as presented in literature [24]. The best model was chosen basing deviance information criterion (DIC) [21].

The type I assumes that the two unstructured effect υ_i and ψ_t interact. Type II combines the structured temporal main effect γ_t and the unstructured spatial effect υ_i. Type III combines the unstructured temporal ψ_t and spatially structured main effect u_i. Finally, type IV is the most complex type of interaction, it assumes the spatially and

Table 1. Interaction types:Parameter interacting and rank of $\mathbf{R}_\delta$

Type of interaction	structure matrix	Rank
Type I interaction	$\mathbf{R}_\delta = \mathbf{R}_\upsilon \bigotimes \mathbf{R}_\psi = I \bigotimes \mathbf{I} = \mathbf{I}$	nT
Type II interaction	$\mathbf{R}_\delta = \mathbf{R}_\upsilon \bigotimes \mathbf{R}_\gamma$	$n(T-2)$ for RW2
Type III interaction	$\mathbf{R}_\delta = \mathbf{R}_\psi \bigotimes \mathbf{R}_u$	$(n-1)T$
Type IV interaction	$\mathbf{R}_\delta = \mathbf{R}_u \bigotimes \mathbf{R}_\gamma$	$(n-1)(T-2)$ for RW2

temporally structured effects u_i and γ_t. We assigned a gamma distribution with shape equal 0.5 and rate equal to 0.00149 following the approach of Fong et al.(2010) [22]. For the remaining parameters, we assigned prior distributions to scaled precision matrix parameters based on their marginal standard deviations on its diagonal following methods proposed by Sorby and Rue (2013) [18].

In order to investigate whether or not a reduction of malaria was observed compared to overall incidence rate in 2012, we make use of excess probability. The probability that the malaria risk has decreased by c% is calculated as the posterior probability $P(\theta_{it} < (100-c)\%)$. If $|P|$ is large, the set goal is likely reached in that area, while if $|P|$ small, it is very likely not been reached. The $|P|$ is being compared to the set goal, which is the expected reduction in malaria incidence expected by 2015 and 2018.

2.4 Estimation methods

We used Integrated Nested Laplace approximation (INLA) for estimation. The INLA is a deterministic algorithm for Bayesian inference and is designed for latent Gaussian models and spatial models. Bayesian estimation using the INLA methodology takes much less time as compared to estimation using Markov Chain Monte Carlo Methods (MCMC). [17]

We performed a sensitivity analysis on variety of model formulations for the latent level due to inherent issues that come with each formulation. It is well known from literature that in BYM model, the spatially structured component cannot be seen independently from unstructured component. As an alternative model BYM2 improves parameter the control on those parameters, allowing the parameter to be seen independently from each other [18]. We fitted both models (BYM and BYM2) using the same priors. Results from both models were similar.

3 Results

The results are presented into two parts. The first part provides summary descriptive statistics of malaria cases and estimates from the fitted spatio-temporal model ~~per year~~. The second part presents evaluation of Rwanda's malaria policy on the reduction of incidence using the excess probability approach. We introduced formal friendly interpretation and classification based on the excess probability approach for decision making during the malaria pre-elimination phase.

3.1 Malaria cases in Rwanda 2012-2018

Rwanda has experienced an increase in malaria cases from 398,287 cases per year in 2012 to 2,956,337 cases in 2016. However, in the last two years 2017 and 2018 the cases reduced to 1,978,450 and 1,725,522 respectively. Figure 1 shows the trend as overall and desegregated by age groups and sex. The year 2015 and 2016 recorded the highest number of cases, in all age/gender groups.

Fig 1. Malaria cases over time by sex

3.2 Malaria relative risk in Rwanda 2012-2018: BYM

~~We have fitted separate models for each year~~ We have fitted spatio-temporal models for seven years period, taking into account both structured and unstructured random effects (BYM and BYM2 models) as it provides a compromise between spatial correlation and extra heterogeneity over time. Since the results of those models are similar, we present BYM model fitted with type II interaction based on DIC 2.

Table 2. Comparison of models basing on DIC

Model	D	pD	DIC	DICc	WAIC
model.ST1	2848807	284.9672	2849092	2849422	2300753
mod.intI	640735.1	5251.247	645986.3	657421.2	941846.9
mod.intII	40046.5	14499.43	54545.93	91864.38	70889.18
mod.intIII	41886.02	16217.6	58103.63	97675.84	76402.72
mod.intIV	6.674699e+106	6.674699e+106	1.33494e+107	6.674699e+106	1.85816e+2[illegible]

Those models provide the estimates at the smallest available geographical scale, that might be an added value to drive oriented and targeted interventions to control malaria in Rwanda.

Fig 2. ~~Posterior temporal trend for Malaria Relative Risk in Rwanda:unstructured effect $\exp(\phi_t)$(solid line) and temporally structured effect $\exp(\gamma_t)$ (dashed line)~~Posterior temporal effect for malaria relative risk in Rwanda:$\exp(\phi_t + \gamma_t)$ with 95% Credible Interval

Estimates of variances due to random effects are presented in Table 3, the contribution of variance can be summarized, showing that about 50% is explained by a spatial component, and 50% by unstructured component. This is also visible in Figure 3, which presents estimated relative risks for each year, compared with the overall incidence rate year in 2012.

Table 3. Posterior mean and 95% Credibility interval for fixed effect of α

**Year**	**Parameter**	**Estimate**	**SD**	**LL**	**UL**
2012	σ_u^2	0.2703	0.0877	0.1414	0.4822
	σ_v^2	0.2407	0.0278	0.19	0.2991
$Frac_{spatial}$	$Var_u/(Var_u + \sigma_v^2)$	52%			
2013	σ_u^2	0.2696	0.0822	0.1454	0.4657
	σ_v^2	0.2668	0.0309	0.2107	0.332
$Frac_{spatial}$	$Var_u/(Var_u + \sigma_v^2)$	49%			
2014	σ_u^2	0.2942	0.0889	0.159	0.5059
	σ_v^2	0.2775	0.0307	0.2216	0.3421
$Frac_{spatial}$	$Var_u/(Var_u + \sigma_v^2)$	50.5%			
2015	σ_u^2	0.3981	0.1455	0.1925	0.7558
	σ_v^2	0.2928	0.0318	0.2342	0.3594
$Frac_{spatial}$	$Var_u/(Var_u + \sigma_v^2)$	56%			
2016	σ_u^2	0.6749	0.2602	0.3131	1.3203
	σ_v^2	0.3877	0.0392	0.3153	0.4691
$Frac_{spatial}$	$Var_u/(Var_u + \sigma_v^2)$	62%			
2017	σ_u^2	0.4947	0.1602	0.2576	0.8805
	σ_v^2	0.4135	0.0442	0.3326	0.5059
$Frac_{spatial}$	$Var_u/(Var_u + \sigma_v^2)$	53%			
2018	σ_u^2	0.3466	0.1016	0.192	0.5879
	σ_v^2	0.4846	0.0615	0.3735	0.6147
$Frac_{spatial}$	$Var_u/(Var_u + \sigma_v^2)$	41%			
2012-2018	$Frac_{spatial}$				
	$Var_u/(Var_u + \sigma_v^2)$	52%			

SD:Standard Deviation, LL: Lower Level, UL: Upper Level

~~Figure 2 shows an increasing trend for structured effect across the years. Though, the unstructured term shows some fluctuation around one.~~ shows an increasing trend effect for malaria relative risk in Rwanda with 95% Credible Interval over years

In general, spatio-temporal contribution to geographic variability are important, as there is a tendency to see low relative risks in the North-West of Rwanda, and high relative risk in the East and in the South of Rwanda. We observe also a large amount of

Fig 3. Malaria Relative Risk from year 2012 to 2018figure updated

heterogeneity amongst areas, as some of the areas with high relative risk for Malaria are surrounded by areas with low risk (and vice versa). Table 4 shows the number of sectors with RR's within specific intervals.

In 2012, 73.8% (307) of all sectors (416) had RR $<$ 1,thus with a lower than average disease rate, while 18.03% (75) of the sectors RR were above one but below 4, and 5.53% (23) above 4 but below 10. Eleven sectors RR was above ten, including four sectors with a relative risk greater than 15. Those four sectors were in the City of Kigali, with the highest RR observed in Gasabo District Gikomero sector (RR= 19.6, 95% CI = 19.13, 20.05). The two other sectors were in the Southern province Kigoma sector in Nyanza (RR= 19.7, 95% CI= 19.23, 20.25) and Gikonko in Gisagara District with a RR 16.8, 95% CI= 16.42, 17.20). The last one in Eastern province, Nyagatare District, was Nyagatare sector with a (RR= 15.75, 95% CI = 15.51, 16.01). This indicates that the malaria cases are concentrated in few areas, while the disease rate is low in most sectors.

In 2013, there is an increase in the number of sectors with RR ranging between one and four. In fact, the category of (1,4) increased to 22.12% as compared to 2012. In 2014, 36 (8.65%) sectors had RR $>$ 4. For the year 2015, 39.9% sectors have higher RR $>$ 1, and 5.3% of sectors had a RR $>$ 4. In 2016, 40.87% of all the sectors had RR $>$ 1 and 6.97% of sectors had RR $>$ 4. In 2017, 37.98% of sectors had a RR $>$ 1 and 9.13 of sectors had a RR $>$ 4. Similar to previous year, in 2018 37.74% of sectors had RR $>$ 1 and 7.69% of sectors had RR $>$ 4. In conclusion, compared to the overall risk in the year 2012, the risk has increased in later years. In addition, the number of sectors with lower than average risk in the year 2012 has decreased over time.

Table 4. Malaria RR per year as compared to the year 2012

Year	**Malaria RR 2012:2018**				
	**[0,1[**	**[1,4[**	**[4,10[**	**[10,15[**	**[15,24[**
2012	307(73.80%)	75(18.03%)	23(5.53%)	7(1.68%)	4(0.96%)
2013	290(69.71%)	92(22.12%)	25(6.01%)	7(1.68%)	2(0.48%)
2014	278(66.83%)	102(24.52%)	30(7.21%)	3(0.72%)	3(0.72%)
2015	250(60.10%)	144(34.62%)	18(4.33%)	3(0.72%)	1(0.24%)
2016	246(59.13%)	141(33.89%)	27(6.49%)	2(0.48%)	0(0%)
2017	258(62.02%)	120(28.85%)	36 (8.65%)	2(0.48%)	0(0%)
2018	259(62.26%)	125(30.05%)	26(6.25%)	5(1.20%)	1(0.24%)

Fig 4. The Area-specific probabilities of not reaching the target goal of 2015 (reduction of 20% as compared to 2012)figure updated

Fig 5. The area-specific probability of not reaching the target goal of 2018 (reduction of 42% as compared to 2012)figure updated

3.3 Assessment of Malaria policy to reduce incidence in Rwanda

Rwanda Malaria's strategic plan 2012-2018 [5] aimed to reduce malaria incidence by 20 % in 2015 and 42% in 2018. Here results are showing explicitly the probability taking into account spatial uncertainty as it provides local details of the spatial variation of the risk. Figures 4 and 5 present the area-specific probabilities not reaching the target goals. Areas colored red have a high probability (above 80%) of not reaching the target goal, while areas in yellow have high probability (above 80%) of reaching the target goal. For areas in orange, we are uncertain about whether or not the sectors succeeded in achieving the target goals.

At the baseline year 2012, 29.33% (122) and 33.65% (140) of sectors had a high probability (> 0.8) to have smaller than average risk (< 0.58 and < 0.80, respectively). The number of sectors that failed to reach target of 20% reduction increased over the years. Similar to target of 42%, the number of sectors that failed to reach the target increased.

This is due to increased malaria incidence across all the sectors from 2012 to 2016. In 2017 and 2018, the incidence reduced, but not lower than in 2012. While an improvement towards reaching the target in some years is seen for some areas, the improvement did not persist over the entire follow-up period. After intervention of insecticide residual spry (IRS) in 2015, 2016 and, 2017 the sectors of Nyagatare (East North) and Kirehe (East south) displayed reduction incidence. At the same time, we see that in the South-West, while targets were reached in the earlier years, these areas failed to sustain progress. Table 5 shows a summary of the number sectors that did not achieve the targets set out by Rwanda's malaria strategic plan with a certain probability.

Table 5. The sectors that did not achieve reducing the targets

Year	**Target of reducing** 20%				
	**[0,0.2[**	**[0.2,0.4[**	**[0.4,0.6[**	**[0.6,0.8[**	**[0.8,1[**
2012	289(69.47%)	1(0.24%)	4(0.96%)	0(0%)	122(29.33%)
2013	271(65.14%)	4(0.96%)	4(0.96%)	0(0%)	137(32.93%)
2014	258(62.02%)	1(0.24%)	1(0.24%)	4(0.96%)	152(36.54%)
2015	222(53.37%)	4 (0.96%)	0(0%)	5(1.20%)	185(44.47%)
2016	218(52.40%)	3(0.72%)	0(0%)	2(0.48%)	193(46.39%)
2017	236(56.73%)	2 (0.48%)	2(0.48%)	3(0.72%)	175(41.59%)
2018	241(57.93%)	2(0.48%)	2(0.48%)	2(0.48%)	169(40.62%)
	**Target of reducing** 42% **by 2018**				
2012	273(65.62%)	1(0.24%)	1(0.24%)	1(0.24%)	140(33.65%)
2013	250(60.10%)	3 (0.72%)	2(0.48%)	2 (0.48%)	159(38.22%)
2014	235(56.49%)	3(0.72%)	6(1.44%)	4(0.96%)	168(40.38%)
2015	200(48.08%)	2 (0.48%)	0(0%)	0(0%)	214(51.44%)
2016	187(44.59%)	1(0.24%)	2(0.48%)	0(0%)	226(54.33%)
2017	203(48.80%)	6(1.44%)	4(0.96%)	3(0.72%)	200(48.08%)
2018	216(51.92%)	0(0%)	3(0.72%)	4(0.96%)	193(46.39%)

4 Discussion

Spatial data has increased substantially due to the advances in computational tools that allow collection and integration of diverse real-time data sources. This goes in hand with the development of less or complex innovative statistical models to deal with the spatial structure of data in hand [24]. Model-based statistical methods are advantageous in low resource settings for estimating disease risk at health decision-making units as well as properties of uncertainty for survey data [25]. In this paper, this is extended towards the estimation of the probability to reach certain target goals.

In the past, a concern of data quality hampered the use of health facility data as a source of population based statistics. Introduction of web-based information systems for health facility data and implementation of universal health policy improved the completeness and accuracy of data at local areas to the extent of providing ~~sound~~ accurate statistics. This was fueled by the intensive monitoring of sustainable development goals [26] [27]. The data from health facilities in Rwanda are of high quality, though successfully integrating these data into health policy and decision-making throughout the health system is an ongoing challenge. [28].

The spatial modeling analysis for Rwanda malaria data from health facilities suggested an overall increase in relative risk (RR) in almost all sectors of Rwanda from 2012 to 2016, with a slight decrease from 2017 and 2018. The number of sectors with RR > 1 has

increased tremendously, in some sectors the RR was above 10.This implies the increase of malaria incidences in each sector of Rwanda; though a slight change was observed as compare to previous years but was not persistent over years This implies that malaria incidence slightly increased over time in all sectors of Rwanda but the increase was not persistent over years.

The estimated probability of achieving target for malaria reduction showed that, almost half (47.36%) (197) of all sectors failed to meet the target of reducing 42% of malaria incidence by 2018, with 80% or 90% certainty. Contrary to the expectation from the Malaria Strategic plan [5], malaria incidence increased in East, South, Central, and West-south of Rwanda. Those areas of Rwanda are known as high malaria risk zones [5]. This means that the malaria control program should concentrate effort on reducing transmission through preventive interventions such as indoor residual spraying (IRS) and bed-net distribution. In 2013, 2015, 2016 and, 2017 as figure 4 and 5 show a change in the east north (Nyagatare) and east south (Kirehe); the reduction might be due to the IRS intervention that occurred in the same period in those Districts. With 90% probability, 51.92% of sectors reduced malaria incidence as planned; however, those sectors belong in Northern provinces and West-North of Rwanda where malaria cases are often relatively low as compared to other parts of Rwanda. Despite those encouraging success, much work remains to achieve malaria reduction targets for the whole country. Implementing pre-elimination strategies in those sectors should be premature, instead the focus should be implementing malaria control strategies considered consciously.

The results presented here are based only on spatial analysis of malaria cases from health facilities and population distribution, and the database had limited variables that could have been included in the analysis to explain increased relative risk and reason of failing to achieve the target of reducing incidence as planned. We limited our scope on statistical method to evaluate reduction of malaria incidence using an excess probability approach. This approach is relevant tool to assess a health policy and guide the decision makers. It can contribute to improve Malaria surveillance to ensure appropriate intervention in the right place and at the right time. While the current unit of decision for malaria control in Rwanda is a district level, we have shown that there is enough data and statistical tools that should enable malaria control program in Rwanda to use sector level as unit for decision.

A disease like Malaria requires a strong surveillance system that enable a quick response to any changes in Malaria behaviour. Efficient algorithms that can be deployed in response to real-time data collection and make inferences would contribute fast response to potential public health threats. [16]

5 Conclusion

In summary, we recommend the approach of using spatio-temporal models and routinely collected facility-based malaria data to assess the malaria targets related to incidence rate and estimate malaria relative risk at the local area level. This approach enables us to generate maps that provide information about the probability and uncertainty of reaching the target goal, as well as providing information on the spatial contribution to Malaria. The proposed approach is not only limited to malaria data, but it can also be applied in other health care delivery.

This era of sustainable development goals (SDGs), especially SDG 3 and its target 3.3 of ending malaria by 2030, requires a tool like the one presented here for planning, monitoring, and evaluation. The excess probability can be applied to survey or routine data from health facilities. It efficiently uses routine data for permanently monitoring the changes in malaria transmission and evaluation progress towards national targets. Though survey data are important, provided that data quality are high, routinely collected data are collected more frequently and thus provide more timely assessments of health burden. Most of the surveys, take five years to get new evidence, an example of DHS (Demographic and Health survey) and often do not provide estimates at a local level.

Acknowledgments

The authors are grateful to Rwanda Biomedical Center, Malaria and other Parasitic Diseases division, for collaboration and discussions on Rwanda Malaria's strategic plan evaluation approaches. We are grateful for Rwanda malaria resourceful materials shared by Dr. Aimable Mbituyumuremyi, Malaria Division manager and technical support provided by Mr. Hamza Ndabateze, HMIS officer, to extract data from Rwanda health

information system (HMIS).

References

1. WHO. World Malaria report 2015[Internet]. Geneva; 2015. Available from: https://www.who.int/malaria/publications/world-malaria-report-2015/report/en/

2. WHO. World Malaria report 2018 [Internet]. Geneva; 2018. Available from: https://www.who.int/malaria/publications/world-malaria-report-2018/en/

3. World Health Organization, Global Malaria Programme. A framework for malaria elimination [Internet]. WHO Press, World Health Organization. 2017. 100p. Available from: http://apps.who.int/iris/bitstream/handle/10665/254761/9789241511988-eng.pdf?sequence=1

4. Ministry of Health (Rwanda). Rwanda Malaria Strategic Plan (2013-2018) Mid Term Review. Rwanda Biomedical Centre. 2016. Available from: http://www.moh.gov.rw/index.php?id=511

5. Rwanda Biomedical Center(RBC). Rwanda Malaria Strategic Plan 2012-2018. RBC:Kigali, Rwanda. 2012.

6. Tesi M. Africa initiative Discussion Papers Global Warming and Health: The Issue of Malaria in Eastern African's Highlands. Global health. 2011; (2).

7. Ministry of Health (Rwanda). Rwanda third health sector strategic plan (2012-2018). Ministry of Health. 2016. Available from: http://www.moh.gov.rw/index.php?id=511

8. Hemingway, J. et al. Tools and Strategies for Malaria Control and Elimination: What Do We Need to Achieve a Grand Convergence in Malaria? PLoS Biology, 2016. 14(3), pp.1–14

9. Dehnavieh, R., Haghdoost, A., Khosravi, A., Hoseinabadi, F., Rahimi, H., Poursheikhali, A., … Aghamohamadi, S. The District Health Information System

(DHIS2): A literature review and meta-synthesis of its strengths and operational challenges based on the experiences of 11 countries. Health Information Management Journal, 2019.48(2), 62–75. https://doi.org/10.1177/1833358318777713

10. Sahay, S., Sæbø, J. & Braa, J. Scaling of HIS in a global context: Same, same, but different. Information and Organization journal, 2013. 23(4), pp.294–323. Available at: http://dx.doi.org/10.1016/j.infoandorg.2013.08.002.

11. WHO,2018, Malaria surveillance, monitoring and evaluation: A reference manual

12. World Health Organization. Epidemiological approach for Malaria control. World Health Organization, 2015. 2nd edition Available at: https://apps.who.int/iris/handle/10665/96351

13. USAID. President's Malaria initiative Rwanda operational plan financial year 2019. Available: `https://www.pmi.gov/docs/default-source/default-document-library/malaria-operational-plans/fy19/fy-2019-rwanda-malaria-operational-plan.pdf?sfvrsn=3`. Accessed 2019 September 27

14. Emmanuel Giorgi et al. Using non-exceedance probabilities of policy-relavant malaria prevalence thresholds to identify areas of low transmission in Somalia Malaria Journal, 2018. 17, Article number: 88

15. Andrew J Tatem, David L Smith, Peter W Gething, Caroline W Kabaria, Robert W Snow, Simon I Hay. Ranking of elimination feasibility between malaria-endemic countries. Lancet 2010; 376: 1579–91

16. Lawson, A. & Lee, D. Bayesian Disease Mapping for Public Health. Handbook of statistics, 2017. volume 36, Pages 443-481. Available at: http://dx.doi.org/10.1016/bs.host.2017.05.001

17. Carroll, R. et al. Comparing INLA and OpenBUGS for hierarchical Poisson modeling in disease mapping. Spatial and Spatio-temporal Epidemiology, 2015. 14–15, pp.45–54 available at:doi: 10.1016/j.sste.2015.08.001

18. Andrea Riebler and Sigrunn H Sørbye and Daniel Simpson and Håvard Rue. An intuitive Bayesian spatial model for disease mapping that accounts for scaling.

Statistical Methods in Medical Research, 2016. 25,4, pp.1145–1165 available at:doi: 10.1177/0962280216660421

19. Su Yun Kang,Susanna M. Cramb, Nicole M. White, Stephen J. Ball,Kerrie L. Mengersen,2016. Making the most spatial information in health: A tutorial in Bayesian Disease Mapping for areal Data Geospatial health Journal. 2016. 31;11(2):428 available at: DOI: 10.4081/gh.2016.428

20. Besag, J., & Green, P. Spatial Statistics and Bayesian Computation. Journal of the Royal Statistical Society. Series B (Methodological), 1993. 55(1), 25-37.

21. Lesaffre,E. and A. B. Lawson. Bayesian Biostatistics.Statistics in practice. UK:John Wiley & Sons. 2012 Available at:DOI:10.1002/9781119942412

22. Fong, Youyi and Rue, Håvard and Wakefield, Jon. Bayesian inference for generalized linear mixed models. Biostatistics. 2010 11, 3, 397-412 Available at:DOI:10.1093/biostatistics/kxp053

23. Besag, J., York, J., & Mollie (1991) Baysian image restoration with two applications in spatial statistics. Ann. Inst. Statist. Math.1991 43: 1. available at:https://doi.org/10.1007/BF00116466

24. Marta Blangiordo, & Michela Cameletti. Spatial and Spatio temporal Bayesian models with R-INLA. UK:John Wiley & Sons.2015 available at:DOI:10.1002/9781118950203

25. Robert Yankson, Evelyn Arthur Anto & Michael Give Chipeta. Geostatistical analysis and mapping of malaria risk in children under 5 using point-referenced prevalence data in Ghana. Malaria Journal. 2019. 18:67 available at:https://doi.org/10.1186/s12936-019-2709-y

26. Sabella Maina, Pepela Wanjala, David Soti,Hillary Kipruto, Benson Drotid & Ties Boermae. Using health-facility data to assess subnational coverage of maternal and child health indicators, Kenya. Bull World Health organ. 2017.1;95:683:694 available at:95(10):683-694. doi: 10.2471/BLT.17.194399

27. Marie Paul Nisingizwe, Hari S. Iyer, Modeste Gashayija, Lisa R. Hirschhorn, Cheryl Amoroso, Randy Wilson, Eric Rubyutsa, Eric Gaju, Paulin Basinga,

Andrew Muhire, Agne's Binagwaho, Bethany Hedt-Gauthier. Toward utilization of data for program management and evaluation: Quality assessment of five years of health management information system data in Rwanda. Global Health Action. 2014.7 available at: ISSN:1654-9716. doi: 10.3402/gha.v7.25829

28. Karengera Innocent, Robert Anguyo DDM Onzima, Simon-Peter, Philip Govule. Quality and Use of Routine Healthcare Data in Selected Districts of Eastern Province of Rwanda. international journal for public health research 2016.(2):5:13 available at:ISSN:2381-4837

Response to the Reviewer #1

Reviewer #1: Routine data are becoming increasingly important as countries adopt DHIS2 across sub-Sahara Africa. The approaches presented in the paper are important to allow national malaria control programmes assess progress (or lack of it) at sub national units of decision making. I have the following comments.

1. What's the fidelity of the data used: were there facilities that were no reporting in some months or not reporting at all? The authors should summarize the completeness of data (how many facilities reported 12 times in year? How many did not report at all?

Response: All the HCs from 416 administration sectors reported 12 times a year for the period of 7 years (2012-2018). This is due to Data quality assessment (DQA) that is performed routinely, DQA report is submitted immediately to ministry of health and Rwanda Biomedical center within five days after each review for direct action. (http://www.moh.gov.rw/fileadmin/Publications/Health_Data/Rwanda_Data_Quality_Assessment_Version_2016.pdf, page 10 for DQA procedures).

Data quality assessment is routinely performed on the data, resulting in fully completeness in the data.

2. Clarify on the manuscript if all data used was lab confirmed

Response: All malaria cases used in this study were lab confirmed (inserted in the manuscript). Over 95% of malaria cases reported are lab confirmed (source: Malaria operational plan FY 2018, President's malaria initiative)

3. Why was the sector used as the unit of analysis? is this the unit of decision used by the national malaria control programme?

Response: Currently the unit of decision used by national malaria control program is a district since it is a statistics domain in surveys such as Demographic health survey (DHS), we used sector as a value added since it is lower level to District. We are proving that we have enough data and statistical tools that would enable malaria control program to use sector level as unit for decision.

4. Often individuals are assigned based on the health facility they attend as opposed to their residential locations in DHIS2. This means the risk in certain sector is not the actual risk because it's based on patients from that sector and the neighbouring sectors. Can the authors elaborate in the Rwanda case and how they handle this issue?

Response: In Rwanda the individuals are assigned to health facility basing to their residential locations in DHS2. Each sector has at least one Health Center that serves all individuals

residents of that sector. All Health centers are equally equipped; therefore, it is unlikely that someone will attend a Health center from another sector rather than the one of his/her location.

5. How were neighbouring areas defined? (queen, rook, based on distance etc.?) and why the choice? It has been shown different choices yield different results

Response: The neighboring areas were defined basing on queen method, after making sensitivity analysis there was no difference in terms of findings disregards of methods used (between queen, rook, nearest distance etc)

6. Likewise, why the BYM model? they are several other choices (Besag, Besag2, BYM2, Leroux CAR, proper CAR etc.) BYM2 handles identifiability and scaling better

Response: We performed sensitivity analysis using Besag, , BYM2, , and BYM2, the was no differences in terms of estimates.

7. The choice of priors and justification is not provided

Response: Sensitivity analysis

We have compared the posterior estimates using different priors and there was no changes in estimates.

8. Why were different models fitted by year instead of a spatio-temporal model to also account for temporal correlation and where necessary space-time interactions?

Response: This is indeed a valid point. We had started with the spatial models as an exploration of the data, but it is better to take into account temporal correlation and space-time interactions. Therefore, the models in the paper have now been changed, and different types of spatio-temporal model have been investigated. This is included in the revised manuscript in Sections 3.2 , table 2and XX.

9. The first two paragraphs of 3.2 should be in the methods section

Response: In these paragraphs, we discuss the results of the models. Thus, it is not clear whether or not we should move this section to the methods section. If required, we can indeed move these paragraphs to the methods section, but we have now kept them in the results section. Note that the first paragraph has been changed considerably, because of the changes in the methodology.

10. Why were there no covariates used to assist in the estimation process?

Response: We did not have covariates in our dataset. However, we are also more interested on evaluating if the target set were achieved or not using exceedance probability approach. The malaria control program has set its targets basing on reduction percentage of overall cases in Rwanda. We have included this comment in the discussion section, since it would indeed be

interesting and important to see how covariates can explain the differences in exceedance probabilities.

11. Are the data used (as indicated) to model and accompanied code provided? Coukld not find the URL to either of the two

Response: The data and codes will be provided as supplementary materials after manuscript is accepted for publication.

Response to the Reviewer #2

Reviewer #2: Review comments

The paper addresses an important issue on using population data and clinical routine malaria data for decision making in control/elimination of malaria. The paper uses a model based approach in the analysis and mapping of malaria incidence in Rwanda, for the period from 2012 to 2018.

A) Minor comments
a. Abstract: specify the SDG number that is being referred to there.

Response: added “goal 3, target 3.3”

b. Abstract: “The results showed an increase of risk of malaria and 47.36% of sectors in Rwanda” is not very clear. Is this increase national or in specific sectors only?

Response: We have revised model by fitting spatio-temporal model, thus the overall increase was observed over the time and we noticed that some specific sectors had continuously increased malaria incidence. The updated results are more explicitly.
c. Line 9:10, do the authors mean “categorised” when they mention “situated”?

Response: Yes, this is exactly what we meant. We have now changed the wording to not confuse the reader.

d. Line 14: Most countries “placed”….

Response: corrected (place changed to placed)
e. Line 36 – 37: …finally increasing %... use “percentage” instead of the symbol.

Response: corrected percentage added and replaced %

f. Line 45: For the “epidemiological”….

Response: corrected epidemiological replaced epidemical

g. Line 46 – 47: Re-write to make it clear.

Response: We have rephrased this sentence.

We now write: "HMIS enables to avail of data in one platform from all the health facilities in Rwanda for analysis and use for evidence-based strategies"

h. Line 54 – 55: …. Patterns in low areas transmission should change to …. "patterns in low transmission areas".

Response: We have changed this as suggested.

i. Line 91 – 92: Can authors specify what percentage of the total was not included in the final analysis?

Response: This percentage is added and the sentence is rephrased for clarification.

We now write:

"some cases of under five were not desegregated by sex thus were excluded in the analysis"

j. Line 97 – 102: Authors need to indicate by properly subscripting the count for time as rightly done for geographic areas in the same paragraph.

Response: The years corresponding to the indices are now included: (i=2012, 2013,2014,…..,t)

k. In Table 1, can the authors add footnotes (or in the caption) to explain what SD, LL and UL are?

Response: This has been added in the table.

SD: Standard Deviation, LL: Lower Level, UL: Upper level

l. Line 228: Change "sound statistics" to "accurate statistics".

Response:This has been changed to accurate statistics

m. Line 234 – 236 is not clear in the current form.

Response: We have rephrased the sentence

"This implies that malaria incidence slightly increased over time in all sectors of Rwanda but the increase was not persistent over years"

into

“This implies the increase of malaria incidences in each sector of Rwanda; though a slight change was observed as compare to previous years but was not persistent over years”

n. Line 237: This in the current form is misleading. Should it be reading: .... almost half (47.36%) of all sectors....

Response: This has been rephrased

(47.36%) replaced 47.36% (197)

o. On lines 246 – 249, what threshold is being looked at here? It’s not coming out very clearly.

Response:

We have added explanation, line 32-35,

p. Figures are not (properly) captioned, making it difficult to follow or align the text to the Figures.

Response: The figures were revised and improved based on the spatio-temporal models

i. The Figure (Fig1_Desc2), titled “The under-five Malaria 2012 0 2018” with no sex specified seems redundant as it is adding very little information. The information presented in the graph can be explained in the text.

Response: The figure 1_Desc2 was removed

ii. The Y-axis on Fig1_Desc5 “Overall malaria per year” is misleading. The Figure needs to be re-done to properly convey the correct information.

Response: The trend graph was fitted, Fig1_Desc5 was replaced

iii. The legends in Figures 2 – 4 should be properly positioned, interfering with maps currently.

Response: This is corrected

q. Go through the manuscript to correct typos and grammatical errors.

Response: We have gone over the manuscript and corrected some typos and grammatical errors.

B) Major comments
a. The language in the current form of the paper still needs extensive editing to make things clear.
i. Abbreviations are not properly defined throughout the document i.e. figure 3 instead of Figure 3 etc.

ii. Capitalisation is not properly used throughout the document.
iii. Several sentences are not very clear as indicated in the minor comments above.
iv. Several places missing commas and full stops – distorting the message
v. Inappropriate tenses used.
vi. Inappropriate use of directions i.e. “east north” as indicated on lines 237 - 250 instead of “North east”

Response: This has been corrected

b. Lines 119 – 122: Authors indicate that they use Bayesian methods for the analysis, and. Have taken the time to explain both the data and process models. However, conspicuously missing are details on the priors used in model fitting.

Response: We have now included all the details with respect to the priors

c. Line 132 – 136: Authors introduce the concept of calculating policy relevant threshold. Three issues arise here.
i. How is the threshold “c” determined or reached at? This is not clearly explained in the document. For the reader to understand the policy relevant goals, determination of these thresholds needs to be clearly explained.

Response: The ministry of Health in Rwanda through Health sector strategic plan III set targets to reduce malaria incidence from 26/1000 baseline in 2011 to 20/1000 in 2015 and 15/1000 in 2018 (http://www.moh.gov.rw/fileadmin/templates/Docs/HSSP_III_FINAL_VERSION.pdf , page 39. Table 15. Baseline and targets for malaria) Those targets are estimated basing on historical data and predicted impact of new interventions to be implemented.

ii. In line 135, authors claim that: “If |P| is large, the set goal is likely reached in that area.” What is |P| being compared to?

Response: |P| is being compared to the set goal, which is the expected reduction in malaria incidence by 2015 and 2018.

iii. In P(Theta_it < (100 - c)%), is the relative risk based on the observed data? If so, this has to be explicitly stated.

Response: The relative risk based on the observed data and expected data. It is now explained on line 123 to 125.

d. The authors have 7 years worthy of data. Enough data to enable a proper spatio-temporal model. However, in lines 157 – 159, they indicate that they have fitted separate models for each year. I find this problematic in the sense that they are underutilising the data and not fully leveraging the information therein. A spatio-temporal model will enable borrowing strengths in the data across both space and time, therefore giving a complete picture of how malaria incidence has changed since the base year of 2012. The model fitted currently has huge

implications on the conclusions authors draw in the paper. I comment on this in later sections below.

Response: We agree that it is better to use a spatio-temporal model. We have revised the model to spatio-temporal model instead of fitting separate models per year. Both methodology and results have been updated accordingly.

e. In Tables 2 and 4 and lines 174 – 192, authors present malaria relative risk per year as compared to base year of 2012. They group RR into 5 groups: 0-1, 1 – 4, 4 – 10, 10 – 15, and 15 – 24. They use square braces. This presents several challenges in the sense that:

i. The mathematical/statistical meaning of these braces means that these groups are not distinct, meanwhile in text, these groups of RR are presented as distinct. Authors should not [0, 1] and [0, 1) will mean two different things.
ii. Again, for example, column one has [0, 1] and column two has [1, 4]. Does this mean that these two RR groups contain both the 1? This is same for all the groups and it is misleading.
iii. More confusing is the fact that in the text, they resort to using round braces. For reasons in point 1 above, this becomes more confusing.
iv. Therefore lines 184 – 192 need to be re-written with correct presentation of Table 2.
f. In section 3.3, lines 193 - 215 authors present an "assessment of Malaria policy to reduce incidence in Rwanda." With this goal of analysis in mind, it makes more sense to use spatio-temporal model, so that the available data take into account the trends leading up to the target year (2015 and 2018) for the target non-exceedance thresholds of 20% and 42% respectively. See comment in point (d) above.

Response: We have revised the model to spatio-temporal model instead of fitting separate models per year.

g. Lines 203 – 205: The authors should endeavor to quantify this increase, for it to be helpful and relevant to policy makers.

Response: With Spatio-temporal model, we have estimated the increase over time and it is incorporated in new results. This is now indeed more relavent to policy makers.

h. Lines 206 – 209 should be re-written to properly convey the message contained in there. More importantly, the claims raised in these lines can be affirmed by using a proper spatio-temporal analysis in relation to the concerns raised in points (d) and (f) above.

Response: rephrased with new results that include Spatio-temporal model

i. Line 237 – 239. Authors claim that almost half (47.36%) of the sectors did not meet the targets with 80 or 90% certainty. What would be helpful is for the authors to show clearly each of these certainties on map. See, for example:
i. Giorgi et. al. (2018), Using non-exceedance probabilities of—relevant malaria prevalence thresholds to identify areas of low transmission in Somalia. Malar J. 2018;17:88.

ii. Macharia et. al. (2019), Spatio-temporal analysis of Plasmodium falciparum prevalence to

understand the past and chart the future of malaria control in Kenya

iii. Yankson et. al. (2019), Geostatistical analysis and mapping of malaria risk in children under 5 using point-referenced prevalence data in Ghana

Response: Thanks for this information. We reviewed the papers and refences were included in the paper. We also included maps of uncertainty in the manuscript.

j. In their discussion, on lines 250 – 251, authors mention that “Implementing pre-elimination strategies in those sectors should be considered consciously.” With the the incidence presented here, it’s not proper for the authors to start talking about pre-elimination. The message to policy implementers should rather be to focus on control strategies at this point.

Response: We rephrased this to

“Implementing pre-elimination strategies in those sectors should be premature, instead much focus should be implementing malaria control strategies.”

k. On lines 258 – 259, authors mention that “It can contribute to improve Malaria surveillance to ensure appropriate intervention in the right place and most needed time.” This is very correct, but based on the statistical analysis presented here, authors should be cautious in their statements on conclusions made. A proper spatio-temporal analysis would be required to make this conclusion on time.

Response: A proper Spatio-temporal model was now fitted to take time into consideration.
