# Supplementary material for: Bayesian spatio-temporal modeling of malaria risk in Rwanda
Source: PLoS One. 2020 Sep 10;15(9):e0238504. doi: 10.1371/journal.pone.0238504 (PMC7482939; doi:10.1371/journal.pone.0238504)
Supplement: Attachment — Submitted filename: Response to Reviewers-Final.docx [file pone.0238504.s005.docx]

**Response to the Reviewer #1**

Reviewer #1: Routine data are becoming increasingly important as countries adopt DHIS2 across sub-Sahara Africa. The approaches presented in the paper are important to allow national malaria control programmes assess progress (or lack of it) at sub national units of decision making. I have the following comments.

1. What’s the fidelity of the data used: were there facilities that were no reporting in some months or not reporting at all? The authors should summarize the completeness of data (how many facilities reported 12 times in year? How many did not report at all?

Response: All the HCs from 416 administration sectors reported 12 times a year for the period of 7 years (2012-2018). This is due to Data quality assessment (DQA) that is performed routinely, DQA report is submitted immediately to ministry of health and Rwanda Biomedical center within five days after each review for direct action. (<http://www.moh.gov.rw/fileadmin/Publications/Health_Data/Rwanda_Data_Quality_Assessment_Version_2016.pdf>, page 10 for DQA procedures).

Data quality assessment is routinely performed on the data, resulting in fully completeness in the data.

2. Clarify on the manuscript if all data used was lab confirmed

Response: All malaria cases used in this study were lab confirmed (inserted in the manuscript). Over 95% of malaria cases reported are lab confirmed (source: Malaria operational plan FY 2018, President’s malaria initiative)

3. Why was the sector used as the unit of analysis? is this the unit of decision used by the national malaria control programme?

Response: Currently the unit of decision used by national malaria control program is a district since it is a statistics domain in surveys such as Demographic health survey (DHS), we used sector as a value added since it is lower level to District. We are proving that we have enough data and statistical tools that would enable malaria control program to use sector level as unit for decision.

4. Often individuals are assigned based on the health facility they attend as opposed to their residential locations in DHIS2. This means the risk in certain sector is not the actual risk because it’s based on patients from that sector and the neighbouring sectors. Can the authors elaborate in the Rwanda case and how they handle this issue?

Response: In Rwanda the individuals are assigned to health facility basing to their residential locations in DHS2. Each sector has at least one Health Center that serves all individuals residents of that sector. All Health centers are equally equipped; therefore, it is unlikely that someone will attend a Health center from another sector rather than the one of his/her location.

5. How were neighbouring areas defined? (queen, rook, based on distance etc.?) and why the choice? It has been shown different choices yield different results

Response: The neighboring areas were defined basing on queen method, after making sensitivity analysis there was no difference in terms of findings disregards of methods used (between queen, rook, nearest distance etc)

6. Likewise, why the BYM model? they are several other choices (Besag, Besag2, BYM2, Leroux CAR, proper CAR etc.) BYM2 handles identifiability and scaling better

Response: We performed sensitivity analysis using Besag, , BYM2, , and BYM2, the was no differences in terms of estimates.

7. The choice of priors and justification is not provided

Response: Sensitivity analysis

We have compared the posterior estimates using different priors and there was no changes in estimates.

8. Why were different models fitted by year instead of a spatio-temporal model to also account for temporal correlation and where necessary space-time interactions?

Response: This is indeed a valid point. We had started with the spatial models as an exploration of the data, but it is better to take into account temporal correlation and space-time interactions. Therefore, the models in the paper have now been changed, and different types of spatio-temporal model have been investigated. This is included in the revised manuscript in Sections 3.2 , table 2and XX.

9. The first two paragraphs of 3.2 should be in the methods section

Response: In these paragraphs, we discuss the results of the models. Thus, it is not clear whether or not we should move this section to the methods section. If required, we can indeed move these paragraphs to the methods section, but we have now kept them in the results section. Note that the first paragraph has been changed considerably, because of the changes in the methodology.

10. Why were there no covariates used to assist in the estimation process?

Response: We did not have covariates in our dataset. However, we are also more interested on evaluating if the target set were achieved or not using exceedance probability approach. The malaria control program has set its targets basing on reduction percentage of overall cases in Rwanda. We have included this comment in the discussion section, since it would indeed be interesting and important to see how covariates can explain the differences in exceedance probabilities.

11. Are the data used (as indicated) to model and accompanied code provided? Coukld not find the URL to either of the two

Response: The data and codes will be provided as supplementary materials after manuscript is accepted for publication.

**Response to the Reviewer #2**

Reviewer #2: Review comments

The paper addresses an important issue on using population data and clinical routine malaria data for decision making in control/elimination of malaria. The paper uses a model based approach in the analysis and mapping of malaria incidence in Rwanda, for the period from 2012 to 2018.

A) Minor comments
a. Abstract: specify the SDG number that is being referred to there.

Response: added “goal 3, target 3.3”

b. Abstract: “The results showed an increase of risk of malaria and 47.36% of sectors in Rwanda” is not very clear. Is this increase national or in specific sectors only?

Response: We have revised model by fitting spatio-temporal model, thus the overall increase was observed over the time and we noticed that some specific sectors had continuously increased malaria incidence. The updated results are more explicitly.
c. Line 9:10, do the authors mean “categorised” when they mention “situated”?

Response: Yes, this is exactly what we meant. We have now changed the wording to not confuse the reader.

d. Line 14: Most countries “placed”….

Response: corrected (place changed to placed)
e. Line 36 – 37: …finally increasing %... use “percentage” instead of the symbol.

Response: corrected percentage added and replaced %

f. Line 45: For the “epidemiological”….

Response: corrected epidemiological replaced epidemical
g. Line 46 – 47: Re-write to make it clear.

Response: We have rephrased this sentence.

We now write: “HMIS enables to avail of data in one platform from all the health facilities in Rwanda for analysis and use for evidence-based strategies”

h. Line 54 – 55: …. Patterns in low areas transmission should change to …. “patterns in low transmission areas”.

Response: We have changed this as suggested.

i. Line 91 – 92: Can authors specify what percentage of the total was not included in the final analysis?

Response: This percentage is added and the sentence is rephrased for clarification.

We now write:

“some cases of under five were not desegregated by sex thus were excluded in the analysis”

j. Line 97 – 102: Authors need to indicate by properly subscripting the count for time as rightly done for geographic areas in the same paragraph.

Response: The years corresponding to the indices are now included: (i=2012, 2013,2014,…..,t)

k. In Table 1, can the authors add footnotes (or in the caption) to explain what SD, LL and UL are?

Response: This has been added in the table.

SD: Standard Deviation, LL: Lower Level, UL: Upper level

l. Line 228: Change “sound statistics” to “accurate statistics”.

Response:This has been changed to accurate statistics

m. Line 234 – 236 is not clear in the current form.

Response: We have rephrased the sentence

“This implies that malaria incidence slightly increased over time in all sectors of Rwanda but the increase was not persistent over years”

into

“This implies the increase of malaria incidences in each sector of Rwanda; though a slight change was observed as compare to previous years but was not persistent over years”

n. Line 237: This in the current form is misleading. Should it be reading: .... almost half (47.36%) of all sectors....

Response: This has been rephrased

(47.36%) replaced 47.36% (197)

o. On lines 246 – 249, what threshold is being looked at here? It’s not coming out very clearly.

**Response:**

**We have added explanation, line 32-35,**

p. Figures are not (properly) captioned, making it difficult to follow or align the text to the Figures.

Response: The figures were revised and improved based on the spatio-temporal models

i. The Figure (Fig1_Desc2), titled “The under-five Malaria 2012 0 2018” with no sex specified seems redundant as it is adding very little information. The information presented in the graph can be explained in the text.

Response: The figure 1_Desc2 was removed

ii. The Y-axis on Fig1_Desc5 “Overall malaria per year” is misleading. The Figure needs to be re-done to properly convey the correct information.

Response: The trend graph was fitted, Fig1_Desc5 was replaced

iii. The legends in Figures 2 – 4 should be properly positioned, interfering with maps currently.

Response: This is corrected

q. Go through the manuscript to correct typos and grammatical errors.

Response: We have gone over the manuscript and corrected some typos and grammatical errors.

B) Major comments
a. The language in the current form of the paper still needs extensive editing to make things clear.
i. Abbreviations are not properly defined throughout the document i.e. figure 3 instead of Figure 3 etc.
ii. Capitalisation is not properly used throughout the document.
iii. Several sentences are not very clear as indicated in the minor comments above.
iv. Several places missing commas and full stops – distorting the message
v. Inappropriate tenses used.
vi. Inappropriate use of directions i.e. “east north” as indicated on lines 237 - 250 instead of “North east”

Response: This has been corrected

b. Lines 119 – 122: Authors indicate that they use Bayesian methods for the analysis, and. Have taken the time to explain both the data and process models. However, conspicuously missing are details on the priors used in model fitting.

Response: We have now included all the details with respect to the priors

c. Line 132 – 136: Authors introduce the concept of calculating policy relevant threshold. Three issues arise here.
i. How is the threshold “c” determined or reached at? This is not clearly explained in the document. For the reader to understand the policy relevant goals, determination of these thresholds needs to be clearly explained.

Response: The ministry of Health in Rwanda through Health sector strategic plan III set targets to reduce malaria incidence from 26/1000 baseline in 2011 to 20/1000 in 2015 and 15/1000 in 2018 (<http://www.moh.gov.rw/fileadmin/templates/Docs/HSSP_III_FINAL_VERSION.pdf> , page 39. Table 15. Baseline and targets for malaria) Those targets are estimated basing on historical data and predicted impact of new interventions to be implemented.

ii. In line 135, authors claim that: “If |P| is large, the set goal is likely reached in that area.” What is |P| being compared to?

Response: |P| is being compared to the set goal, which is the expected reduction in malaria incidence by 2015 and 2018.

iii. In P(Theta_it < (100 - c)%), is the relative risk based on the observed data? If so, this has to be explicitly stated.

Response: The relative risk based on the observed data and expected data. It is now explained on line 123 to 125.

d. The authors have 7 years worthy of data. Enough data to enable a proper spatio-temporal model. However, in lines 157 – 159, they indicate that they have fitted separate models for each year. I find this problematic in the sense that they are underutilising the data and not fully leveraging the information therein. A spatio-temporal model will enable borrowing strengths in the data across both space and time, therefore giving a complete picture of how malaria incidence has changed since the base year of 2012. The model fitted currently has huge implications on the conclusions authors draw in the paper. I comment on this in later sections below.

Response: We agree that it is better to use a spatio-temporal model. We have revised the model to spatio-temporal model instead of fitting separate models per year. Both methodology and results have been updated accordingly.

e. In Tables 2 and 4 and lines 174 – 192, authors present malaria relative risk per year as compared to base year of 2012. They group RR into 5 groups: 0-1, 1 – 4, 4 – 10, 10 – 15, and 15 – 24. They use square braces. This presents several challenges in the sense that:

i. The mathematical/statistical meaning of these braces means that these groups are not distinct, meanwhile in text, these groups of RR are presented as distinct. Authors should not [0, 1] and [0, 1) will mean two different things.
ii. Again, for example, column one has [0, 1] and column two has [1, 4]. Does this mean that these two RR groups contain both the 1? This is same for all the groups and it is misleading.
iii. More confusing is the fact that in the text, they resort to using round braces. For reasons in point 1 above, this becomes more confusing.
iv. Therefore lines 184 – 192 need to be re-written with correct presentation of Table 2.
f. In section 3.3, lines 193 - 215 authors present an “assessment of Malaria policy to reduce incidence in Rwanda.” With this goal of analysis in mind, it makes more sense to use spatio-temporal model, so that the available data take into account the trends leading up to the target year (2015 and 2018) for the target non-exceedance thresholds of 20% and 42% respectively. See comment in point (d) above.

Response: We have revised the model to spatio-temporal model instead of fitting separate models per year.

g. Lines 203 – 205: The authors should endeavor to quantify this increase, for it to be helpful and relevant to policy makers.

Response: With Spatio-temporal model, we have estimated the increase over time and it is incorporated in new results. This is now indeed more relavent to policy makers.

h. Lines 206 – 209 should be re-written to properly convey the message contained in there. More importantly, the claims raised in these lines can be affirmed by using a proper spatio-temporal analysis in relation to the concerns raised in points (d) and (f) above.

Response: rephrased with new results that include Spatio-temporal model

i. Line 237 – 239. Authors claim that almost half (47.36%) of the sectors did not meet the targets with 80 or 90% certainty. What would be helpful is for the authors to show clearly each of these certainties on map. See, for example:
i. Giorgi et. al. (2018), Using non-exceedance probabilities of—relevant malaria prevalence thresholds to identify areas of low transmission in Somalia. Malar J. 2018;17:88.

ii. Macharia et. al. (2019), Spatio-temporal analysis of Plasmodium falciparum prevalence to understand the past and chart the future of malaria control in Kenya

iii. Yankson et. al. (2019), Geostatistical analysis and mapping of malaria risk in children under 5 using point-referenced prevalence data in Ghana

Response: Thanks for this information. We reviewed the papers and refences were included in the paper. We also included maps of uncertainty in the manuscript.

j. In their discussion, on lines 250 – 251, authors mention that “Implementing pre-elimination strategies in those sectors should be considered consciously.” With the the incidence presented here, it’s not proper for the authors to start talking about pre-elimination. The message to policy implementers should rather be to focus on control strategies at this point.

Response: We rephrased this to

“Implementing pre-elimination strategies in those sectors should be premature, instead much focus should be implementing malaria control strategies.”

k. On lines 258 – 259, authors mention that “It can contribute to improve Malaria surveillance to ensure appropriate intervention in the right place and most needed time.” This is very correct, but based on the statistical analysis presented here, authors should be cautious in their statements on conclusions made. A proper spatio-temporal analysis would be required to make this conclusion on time.

Response: A proper Spatio-temporal model was now fitted to take time into consideration.
