# Supplementary material for: Bayesian spatio-temporal modeling of malaria risk in Rwanda
Source: PLoS One. 2020 Sep 10;15(9):e0238504. doi: 10.1371/journal.pone.0238504 (PMC7482939; doi:10.1371/journal.pone.0238504)
Supplement: Attachment — Submitted filename: Response to the reviewer.docx [file pone.0238504.s008.docx]

**Response to the reviewer’s comments**

**Manuscript Title: Spatio-temporal modeling for malaria risk in Rwanda**

**Date: April 17, 2020, 2020**

a. Language in the revised form of the paper still falls shy of the required standard. As advised previously, several things are still glaringly evident such as, among others. I strongly advise the authors to seek services of a professional editing service.

Response:

Thank you for your advice, the manuscript was reviewed independently by 3 English Language native speakers

i. Several places missing commas and full stops – distorting the message

Response:

Thank you, it is corrected. The commas, full stops and other punctuations were considered.

ii. Inappropriate tenses used.

Response:

Thank you, It is corrected

iii. Inappropriate use of directions i.e. “east north” as indicated on instead of “North east”.

Response:

Thank you, it is corrected. The appropriate direction was used in revised manuscript.

b. Lines 70 – 71 are confusing, the authors indicate that they use spatial disease mapping techniques, whereas in in methods, they indicate that they are using spatio-temporal methods.

Response:

Thank you for your comment, we have revised line 70-71 to read as follows.

“..in this paper, spatio-temporal methods will be used to investigate the geographical variation of malaria risk.”

c. Lines 145 – 147 What is the justification for using the gamma prior Ga(0.5,0.00149) apart from just copying what Fong et. al. did?

Response:

We have added extra explanation on using gamma prior Ga(0.5, 0.00149) and Sensitivity analysis was also performed

d. Table 2, the authors present a number of things including DICc and WAIC, which they don’t discuss anywhere in the text. Explain what these are.

Response:

We have in the revised version discussed the content in table 2. On page 10, line 192-196

e. Tables 4 and 5, the headers for the Table are wrongly placed, the braces are wrong as well, edit to correct.

We have edited tables 4 and 5 headers as suggested. We have also corrected the braces.

f. There is a disconnect between the paper’s discussion and methods used, hence results found – methods aren’t discussed to highlight how the results and conclusions made have been reached at.

The disconnect that was between the methods and discussion section has been corrected by clearly specifying that “it is spatio-temporal analysis not spatial analysis”

g. I attach the PDF file on which I made a number of comments that will be helpful to the authors to sort out the highlighted issues.

Response:

Thank you very much for all your comments. We very grateful.
