# Supplementary material for: Bayesian spatio-temporal modeling of malaria risk in Rwanda
Source: PLoS One. 2020 Sep 10;15(9):e0238504. doi: 10.1371/journal.pone.0238504 (PMC7482939; doi:10.1371/journal.pone.0238504)
Supplement: Attachment — Submitted filename: Response to the reviewer.docx [file pone.0238504.s009.docx]

**Response to the reviewer’s comments**

**Manuscript Title: Spatio-temporal modeling for malaria risk in Rwanda**

**Date: May 30, 2020**

1. Provide a model selection approach that is not not exclusively based on indices as those used in Table 2. One approach might be to identify models that give more precise and accurate predictions as well as reliable 95% confidence intervals using a hold out sample. I am not convinced that model.intIV has extremely large values for the the chosen indices due to numerical instability..

Response:

Thank you for your advice. Indeed, you are right, the model.intIV did not converge. It was not chosen neither discussed in this manuscript due to convergence issues. We decided to remove the model.intIV from the Table 2 since it is not part of final findings and DIC are not reasonable.

In addition to DIC, sensitivity analysis for model selection, we have added Conditional predictive ordinate, that split data into the two groups. The model is run on y_f_ so that posterior distribution for the parameters p(θ| y_f_ ) is obtained; R-INLA runs the so-called leave one out cross validation ,which assumes that y_f_ =y_i y_c_ =y_i_. The CPO was computed in R-INLA.

2. Provide a more clear explanation of the spatio-temporal models. For example the verb "to combine" is too vague and it is not clear what that mean mathematically. Also, the fact that some covariance matrices have Kroncker products is because the authors have multiplied main spatial and temporal effects (in which case "to combine" means "to multiply")? The authors could provide more explanation of this either in the main manuscript or in the supplementary material, as they prefer.

Response:

This comment is relevant. More clarification was provided on sptio-temporal models, and particularly on Kronecker products. The explanation is provided on lines 140-149.

In fact, there are Kronecker products because there is interaction between space and time. Therefore, the structure matrix is factorized as a Kronecker product of corresponding main effects which interact. The detailed materials are provided in R codes annexed.

Thank you very much for all your comments. We are very grateful.
