# Supplementary material for: Bayesian spatio-temporal modeling of malaria risk in Rwanda
Source: PLoS One. 2020 Sep 10;15(9):e0238504. doi: 10.1371/journal.pone.0238504 (PMC7482939; doi:10.1371/journal.pone.0238504)
Supplement: Attachment — Submitted filename: Response to Reviewer.docx [file pone.0238504.s010.docx]

**Response to Reviewer’s comments**

1. - More explanation about the condition predictive ordinate needs to be provided. Simply mentioning that this available in INLA is not enough and does not provide the reader with enough information to assess the validity of this approach.

Answer:

Thank you for this comment, we have provided more explanation from line 144 to 155 to enable the reader to have enough information and understanding on CPO

2. -The explanation given for the models in Table 1 is not clear. Especially, it is impossible for the reader to understand what is the difference between the models from Type II to Type IV.

Answer:

Thank you, we have provided more explanation on the table 1 by adding three paragraphs. Line 156 to 183. We explained each type of interaction in details to enable the reader to have a good understanding of each type of model interaction (Type I to IV)

3. - Discarding model Type IV because of failure to converge is not a valid justification because it also implies that using a different fitting algorithm may indeed lead to convergence. However, a plausible explanation may also be that the model of Type IV is indeed too complex for the data. The authors should think of how to show evidence that this is indeed the case. In a non-Bayesian context, a natural approach would be to show that the profile likelihood for the covariance parameters is flat and the 95% confidence interval based on that is indeed too wide.

Answer:

Thank you, though model Type IV converged the variance is too small (Stdev: 0.00269982) due to

overspecification. Therefore, it is hard to see the differences as compared to type II.
